# Energy exchange between Nd^3+^ and Er^3+^ centers within molecular complexes†

**DOI:** 10.1039/d4sc03994g

**Published:** 2024-10-18

**Authors:** Diamantoula Maniaki, Annika Sickinger, Leoní A. Barrios, David Aguilà, Olivier Roubeau, Yannick Guyot, François Riobé, Olivier Maury, Laura Abad Galán, Guillem Aromí

**Affiliations:** a Departament de Química Inorgànica i Orgànica, Universitat de Barcelona Diagonal 645 08028 Barcelona Spain aromi@ub.edu; b Institute of Nanoscience and Nanotechnology of the University of Barcelona (IN2UB) Barcelona Spain; c Departamento de Química Inorgánica, Fac. CC. Químicas, Universidad Complutense de Madrid Avda. Complutense s/n 28040 Madrid Spain laabad03@ucm.es; d Univ Lyon, ENS Lyon, CNRS, UMR 5182, Laboratoire de Chimie F69342 Lyon France; e Instituto de Nanociencia y Materiales de Aragón (INMA), CSIC and Universidad de Zaragoza Plaza San Francisco s/n 50009 Zaragoza Spain; f Univ. Lyon, Institut Lumière Matière, UMR 5306 CNRS-Université Claude Bernard Lyon 1, 10 rue Ada Byron F-69622 Villeurbanne Cedex France; g Univ. Bordeaux, CNRS, Bordeaux INP ICMCB UMR 5026 F-33600 Pessac France

## Abstract

Developing controlled and reproducible molecular assemblies incorporating lanthanide centers is a crucial step for driving forward up- and down-conversion processes. This challenge calls for the development of strategies to facilitate the efficient *in situ* segregation of different Ln metal ions into distinct positions within the molecule. The unique family of pure [LnLn′Ln] heterometallic coordination compounds previously developed by us represents an ideal platform for studying the desired Ln-to-Ln′ energy transfer (ET). In this context, we report here the new pure one-step synthetically produced [ErNdEr] (3) complex, which allows for the first time at the molecular level to study the mechanisms behind Nd-to-Er energy transfer. To further assess the photophysical properties of this complex, the analogous [LuNdLu] (1) and [ErLaEr] (2) complexes have also been prepared and photophysically studied. Efficient sensitization *via* the two β-diketones employed as main ligands was probed for both Nd^3+^ and Er^3+^ ions, resulting in highly resolved emission spectra and sufficiently long excited state lifetimes, which allowed further assessment of the Ln-to-Ln′ ET. This intermetallic transfer was first detected by comparing the emission spectra of iso-absorbant solutions and demonstrated by comparing the lifetime values with or without the lanthanide quencher (Er^3+^), as well as with a deep analysis of the excitation spectrum of the three complexes. Thus, a very unique phenomenon was discovered, consisting of a mutual Nd-to-Er and Er-to-Nd ET with no net increase of brightness by any metal; while Nd^3+^ transfers the energy received from the antenna to Er^3+^, the sensitization of the latter results in back-transfer to Nd^3+^ into a non-emissive, thus silent, state.

## Abstract 2

A pure heterometallic coordination complex featuring the [ErNdEr] at the core has unveiled an unprecedented phenomenon of two simultaneous intramolecular energy energy transfer between Nd^3+^ and Er^3+^.

## Graphical abstract

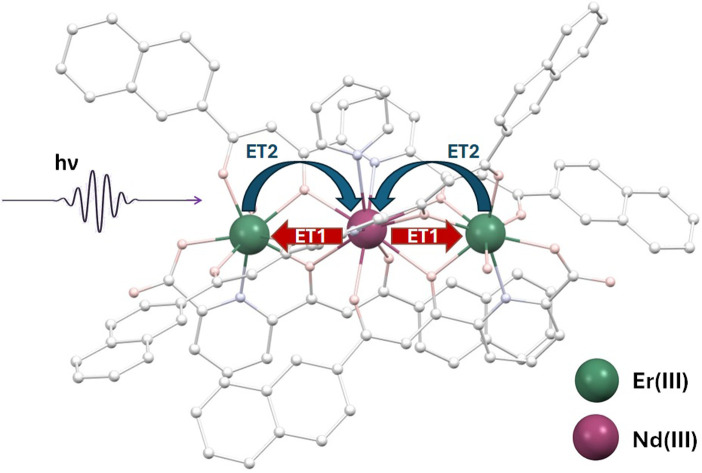

## Introduction

**Fig. 1:**
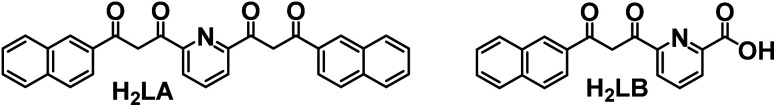
Ligands 2,6-bis-((3-oxo-3-naphthalene-2-yl)propionyl)-pyridine (H_2_LA) and 6-(3-(naphthalene-2-yl)-3-oxopropanoyl)-picolinic acid (H_2_LB).

Lanthanides in their trivalent form (Ln^3+^) are well known for their characteristic optical (and also magnetic) properties. The specific electronic configuration 4f^*n*^5s^2^5p^6^ (*n* = 0–14) when zero-valent, in which the 4f inner shell is well shielded by the 5s and 5p orbitals, gives rise to long-lived excited states with sharp 4f emission bands that span from the near-infrared (NIR) to the visible and UV regions. These optimal photoluminescence properties account for their application in medical imaging,^1–3^ telecommunications,^4–6^ light emitting devices^7^ and solar energy harvesting^8–10^ among other areas.^11^ Ln ions have the major drawback that their intraconfigurational f–f electronic transitions are mostly forbidden by the selection rules, resulting in very low absorption coefficients. This can be overcome by the presence of a light-harvesting antenna which efficiently transfers the absorbed energy to the lanthanide ions, bringing them into excited states. Conjugated organic groups^12,13^ and charge transfer states of d-metal complexes^14–16^ are often presented as the most efficient vectors of lanthanide sensitization. In addition, the electronic configuration of the Ln atoms leads to the presence of 
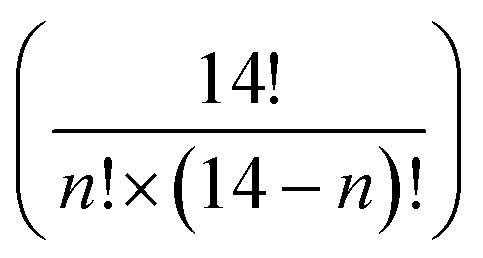
 electronic levels and ladder-like energy diagrams, thus, setting up a perfect platform of possible lanthanide to lanthanide (Ln-to-Ln′, Ln ≠ Ln′) energy transfer events resulting in up- and down-conversion processes.^17^ While these processes have been extensively studied in solids^18–20^ and more recently in nanoparticles,^21–24^ there is a growing interest in exploring their potential at the molecular scale, using stable, well defined and reproducible molecular assemblies.^25^ Regarding up-conversion processes, improvements have been recently obtained with complexes presenting mainly the following pairs: Tb/Yb,^26,27^ Eu/Yb^28^ and Yb/Er.^29–31^ This anti-Stokes luminescence process (lower-to-higher energy transfer) bears significant potential for various applications that require or utilize NIR radiation, including deep-tissue imaging, cancer therapy, nano-thermometry, biosensing, display technologies, and solar cells.^21,32–34^ By contrast, down-conversion (higher-to-lower energy transfer) remains less investigated with the majority of the processes studied in solution.^35^ Probing Ln-to-Ln′ energy transfer (ET) within molecules is challenging because of the necessity to engineer well-defined multi-metallic compounds mixing lanthanide ions with seemingly identical coordination chemistry.^36,37^ Therefore, one-step self-assembly reactions involving different lanthanides often result in mixtures of metal distributions within the molecule^38–41^ and thus, very tedious sequential methodologies, such as covalent linkage of preformed coordination complexes,^36,42–45^ are typically employed to obtain site-selective heterometallic Ln molecules. The synthetic procedures that are thermodynamically controlled stem from the ability to discriminate between the ionic radii of different metals (rLn). Along these lines, we discovered a system capable to coordinate, with remarkable selectivity, two different lanthanide metals by generating two distinct coordination sites, one able to bind the larger ion and the other the smaller one.^46–48^ These structures were observed with heteroleptic complexes using multitopic ligands combining ONO chelates (dipicolinate type) with diketonate moieties. This principle has been successfully replicated with a different architecture thus underlining its great potential.^49^ The new molecular structure was revealed by mixing two ligands (Fig. 1), both with dipicolinate (O, N, O) and diketonate (O, O) units (H_2_LA, 2,6-bis[(3-oxo-3-naphthalene-2-yl)-propionyl]pyridine; H_2_LB, 6-(3-(naphthalene-2-yl)-3-oxopropanoyl)-picolinic acid), with certain combinations of two different Ln(NO_3_)_3_ salts. As a result, both metals are selectively positioned in a [LnLn′Ln] topology. The selectivity presented by these systems, as well as a distance between centers of ∼3.8 Å, allowed us to use this platform to study the intramolecular ET processes. Indeed, we succeeded in studying the Nd-to-Yb ET in the Nd/Yb pair by isolating the [NdYb]^50^ and [YbNdYb]^51^ complexes. We noted that in the case of the trinuclear analogue, the existence of two acceptors per donor dramatically enhances the efficiency of this photophysical process. These findings provide noteworthy examples of down conversion processes in heteronuclear complexes, very little studied so far. The down conversion intramolecular energy transfer has only been presented in the visible region with the Tb/Eu,^52–54^ and Dy/Tb^43^ pairs and, more interestingly, in the NIR region with the Eu/Nd,^55^ Nd/Yb,^50,51,56,57^ Tb/Yb^58^ and Yb/Er pairs.^52,59–61^

This work presents, to our knowledge, the first case of direct energy transfer between Nd^3+^ and Er^3+^ centers in molecular complexes, specifically, in pure heterometallic molecules. This transfer was first observed in glasses in the 1990s,^62,63^ but had not been studied further in molecular systems. Hereby, we synthesized and determined the structure of a new compound, [Er_2_Nd(LA)_2_(LB)_2_(H_2_O)_2_(py)](NO_3_), here termed also [ErNdEr] (3), which effectively promotes intramolecular ET from Er to Nd. The photophysical properties of this complex, as well as those of the previously reported [Lu_2_Nd(LA)_2_(LB)_2_(H_2_O)_2_(py)](NO_3_)^51^ or [LuNdLu] (1) and [Er_2_La(LA)_2_(LB)_2_(H_2_O)_2_(py)](NO_3_)^64^ or [ErLaEr] (2) were thoroughly investigated to best characterize the desired Nd-to-Er ET.

## Results and discussion

### Synthesis

The new complex [Er_2_Nd(LA)_2_(LB)_2_(H_2_O)_2_(py)](NO_3_) (3), which allowed unveiling the phenomenon reported here, was obtained as single crystals from a one-step reaction between the stoichiometric amounts of Er(NO_3_)_3_, Nd(NO_3_)_3_, H_2_LA and H_2_LB in pyridine, following the diffusion of heptane. The chemical process is amenable to a description with a balanced chemical equation that invokes the presence of adventitious water and the association of the released protons with pyridine molecules from the solvent, as the sole base available (eqn (1)):

1 2Er(NO_3_)_3_ + Nd(NO_3_)_3_ + 2H_2_LA + 2H_2_LB +2H_2_O + 9py → [Er_2_Nd(LA)_2_(LB)_2_(H_2_O)_2_(py)](NO_3_) + 8HpyNO_3_

Formation of suitable single crystals of 3 required the addition of one equivalent of CuCl_2_, which does not participate in the reaction but presumably plays a role of a modulator of the crystallization (see ESI† for details).^65^ The formulation of 3 was established by single-crystal X-ray diffraction (SCXRD, see below). The uniqueness of this compound is its strict and well defined heterometallic nature, exhibiting two Er^3+^ atoms per atom of Nd^3+^. Indeed, multinuclear Ln complexes from one step reactions frequently feature quasi-statistical distributions of the different metal types within the molecule, since they have very similar chemical behavior.^38^ In the present case, the scaffold generated by LA^2−^ and LB^2−^ generates coordination sites of two distinct types (the central and the peripheral ones) that favor longer metal-to-donor bond distances in the central location, driving the binding of the larger metal to this position (see structural details below). The purity of the bulk material is supported by C, H, and N microanalysis and inductively coupled plasma (ICP) metal content determination, the latter providing a molar Nd/Er ratio of 0.53. The formulation was also consistent with the response from variable temperature molar paramagnetic susceptibility and variable field magnetization measurements (details in ESI and Fig. S1†), which are ascribed to the presence of one Nd^3+^ and two Er^3+^ ions within a molecule with the molecular mass of 3. The persistence of the architecture of complex 3 in solution was established by mass spectrometry (MS), which unveiled three dominant signals from the trinuclear complex containing its four bridging ligands (Fig. S2–S5†). The absence of any trinuclear moiety with a metal composition other than [ErNdEr] reveals that no metal scrambling occurs.

### Structure

**Fig. 2:**
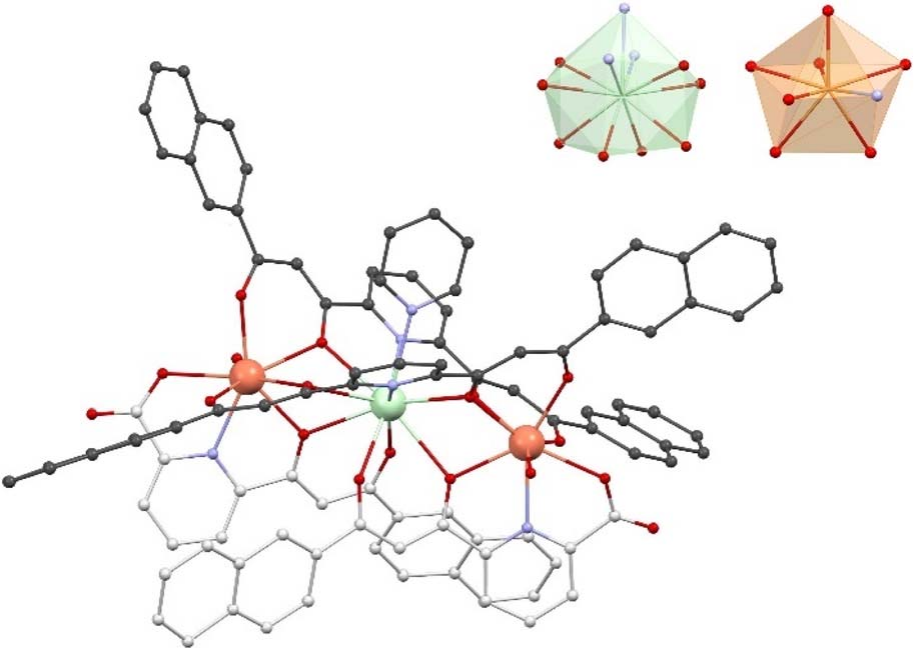
Representation of the cation [Er_2_Nd(LA)_2_(LB)_2_(H_2_O)_2_(py)]^+^ of 3 (also representing those of 1 and 2). The carbon atoms of ligands LA^2−^ and LB^2−^ are darker and lighter gray, respectively. Er is orange, Nd is green, O is red, N is purple and C is gray. H atoms are not shown. The inset shows the coordination polyhedrons of Nd^3+^ and Er^3+^, using the same color codes (both Er^3+^centers have very similar polyhedrons, despite not being crystallographically equivalent, thus, only one is shown).

Detailed information on the molecular structure of 3 was obtained from SCXRD data collected at 100 K. Its crystal lattice belongs to the triclinic space group *P*1̄. The asymmetric unit is made of one formula unit of 3 and 10 molecules of pyridine. The main [ErNdEr] complex (Fig. 2 and S6†) features a quasi-linear trimetallic Er⋯Nd⋯Er array (angle of 174.17°) with the metals bridged together by two *μ*_3_-LA^2−^ and two μ-LB^2−^ ligands that chelate them with two types of pockets, bis-β-diketonate units (O, O) and dipicolinate-like sites (O, N, O). The intramolecular Er⋯Nd separations are 3.944 and 3.947 Å, while the Er⋯Er distance within the complex is 7.881 Å. Interestingly, a shorter intermolecular Er⋯Er distance (6.059 Å) was observed between the symmetry equivalent complexes, associated by complementary H-bonding interactions *via* their water ligands (Fig. S7†). In addition to this intermolecular interaction, each complex interacts with a second one through a π⋯π interaction involving the central pyridine groups of two LB^2−^ ligands (Fig. S8†). Each molecule is related to a third complex through another set of π⋯π contacts involving a total of seven short distances between sp^2^ C-atoms from LA^2−^ ligands (Fig. S9†).

The coordination environment of each Er^3+^ ion is made up of one (O, N, O) and two (O, O) coordination pockets in addition to one molecule of H_2_O, thus featuring a coordination number (CN) of 8. The Nd^3+^ centers exhibit two (O, O) and two (O, N, O) chelates and one pyridine ligand, yielding CN 11. The program SHAPE^66^ was used to determine the ideal polyhedron that represents best the coordination geometry around each metal (Table S4†). For Er^3+^ it is a biaugmented trigonal prism with normalized distances (in a 0 to 100 scale) of 1.639 (Er1) and 1.487 (Er2). Nd is best represented by the figure of a capped pentagonal antiprism, with a calculated distance from it of 6.267. The bond distances to the metals were compared using the Ln–O average distances at each center. These average values are 2.32/2.30 and 2.60 Å for Er1/Er2 and Nd, respectively. Thus, the bond distances to the central metal are about 15% longer than to the peripheral metals.

### Photophysical properties

To assist the investigation of the intermolecular Nd-to-Er energy transfer within the [ErNdEr] (3) molecule, the analogous [LuNdLu] (1) and [ErLaEr] (2) complexes were also studied under the same conditions. The experiments were performed in dilute solutions (10^−4^ M) of MeOH-d_4_ and DMSO-d_6_ (1 : 1) to avoid intermolecular transfers both at room temperature and at 77 K. Studies in the solid state were also performed to ascertain that the complexes in solution coincide with those described by SCXRD (see below). The main photophysical data extracted from this study are compiled in Table 1.

**Table 1:** Summary of the main photophysical data of complexes 1, 2 and 3 in a mixture of deuterated MeOH -*d*_4_:DMSO-*d*_6_ (1 : 1) at room temperature and 77 K (between brackets)

Compound	*λ*_em_ (nm)	*τ*_obs_ (μs)	*Φ*_ET_ b
LuNdLu (1)	1058	3.99 (3.70)	—
ErLaEr (2)	1530	2.85 (3.50)^a^	—
ErNdEr (3)	1058	1.00 (1.80)	0.75 (0.51)
	1530	1.90 (2.30)^a^	0.33 (0.34)

a Measured with an external resistance of 1 kΩ.

b Following eqn (2).

In previous studies, we established the energy of the triplet excited state of ligands H_2_LA and H_2_LB to be about 18 950 cm^−1^ and 19 050 cm^−1^, respectively.^51^ Both energies are sufficiently high to sensitize the ^4^F_3/2_ state of Nd^3+^ centered at, approximately, 11 260 cm^−1^ and the ^4^I_11/2_ or the ^4^I_13/2_ states of Er^3+^ at 10 150 cm^−1^ and ∼6500 cm^−1^, respectively (see below). Therefore, emissions at 1058 nm and 1530 nm can be expected from compounds 1 and 2, respectively. These complexes were thus first investigated to confirm the mentioned antenna effect and to study their luminescence properties.

**Fig. 3:**
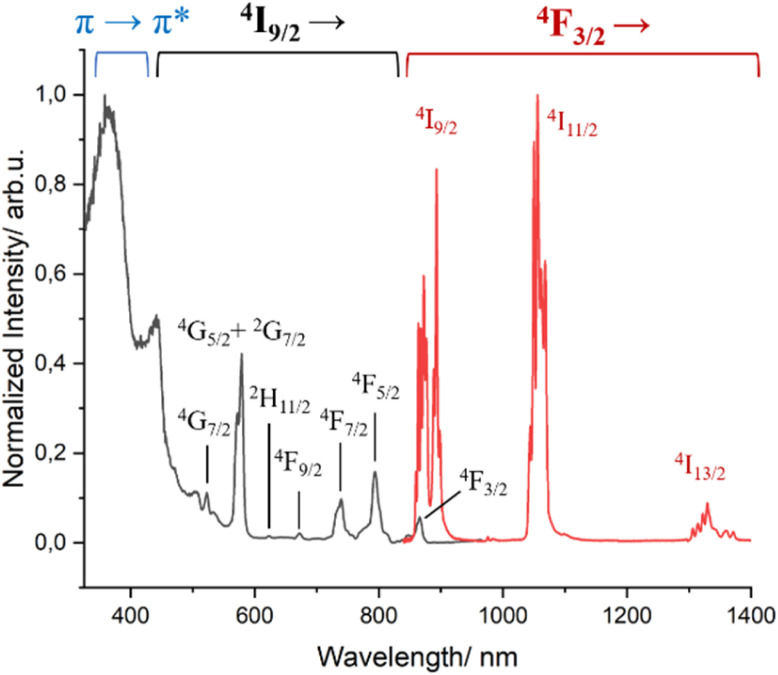
Excitation (*λ*_em_ = 1058 nm, black trace) and emission (*λ*_exc_ = 400 nm, red trace) spectra of complex 1 in MeOH-d_4_ : DMSO-d_6_ (1 : 1) at 77 K.

The photophysics of complex 1 was previously studied in non-deuterated solutions.^51^ We thus now repeated the measurements under deuterated conditions to compare its properties with those of complex 3 (Fig. 3 and S10†). As expected, upon excitation in the ligand transition, 1 presents the characteristic Nd^3+^ transitions at ∼880 nm, ∼1058 nm and ∼1530 nm assigned to ^4^F_3/2_ → ^4^I_*J*_ (*J* = 9/2, 11/2, 13/2), respectively. An increase of the excited state lifetime at room temperature was observed (3.99 μs *vs.* 1.8 μs) due to a decrease of the non-radiative relaxation, especially C–H and O–H vibrational quenching, by the use of deuterated solvents.^67,68^ The excitation spectrum (*λ*_em_ = 1056 nm) at 77 K shows two types of sensitization of the excited states of Nd^3+^, the mentioned antenna effect by energy transfer from the triplet states of the ligands (∼375 nm) in addition to that caused by direct excitations as the f–f transitions (properly assigned in Fig. 3).

**Fig. 4:**
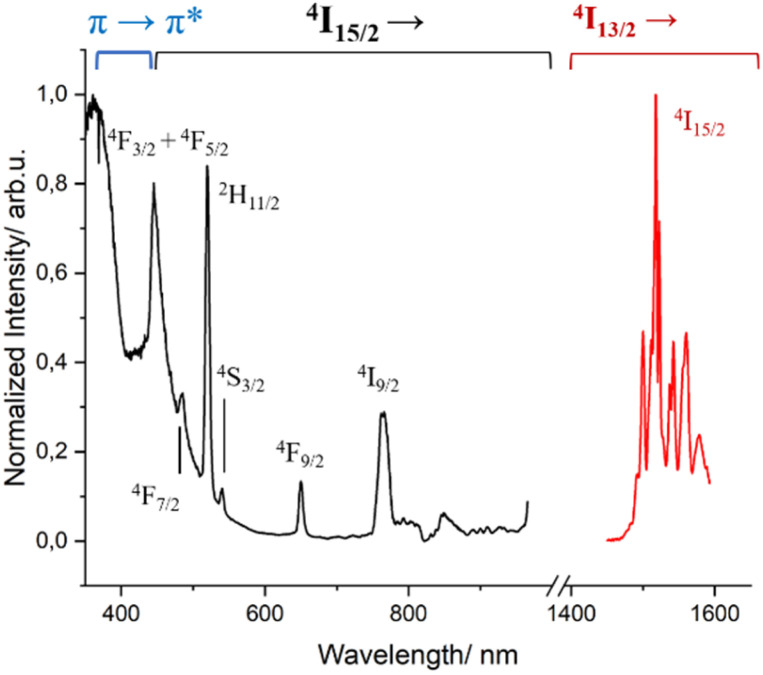
Excitation (*λ*_em_ = 1530 nm, black trace) and emission (*λ*_exc_ = 400 nm, red trace) spectra of complex 2 in MeOH-d_4_ : DMSO-d_6_ (1 : 1) at 77 K.

Complex 2 presents the characteristic broad emission band of the ^4^I_13/2_ → ^4^I_15/2_ transition of Er^3+^ centered at 1530 nm (Fig. S10†), often solely observed in coordination complexes.^69,70^ When this spectrum was measured at 77 K, the intricate fine splitting of the ^4^I_15/2_ state could be observed (Fig. S11†) in addition to the disappearance of the “hot bands” at higher energy of the emissive ^4^I_13/2_ state.^71^ The same fine splitting was observed in frozen solution, indicating that the first coordination environment of Er^3+^ observed in the solid state is kept in solution (Fig. S10 and S11†). The excited-state lifetime decay measured at 1530 nm was fitted with a mono exponential function both at room temperature and 77 K, giving values of 2.85 μs and 3.50 μs, respectively. As seen, lowering the temperature has only a subtle positive effect on the lifetime value by slightly minimizing the non-radiative deactivation. These values are in the typical range of other β-diketonate-based Er^3+^ complexes.^69,72,73^ The respective excitation spectra in solution at low temperature show, as for complex 1, the antenna effect and the effect of the direct excitation of the ^4^I_15/2_ state of Er^3+^, as depicted in Fig. 4.

**Fig. 5:**
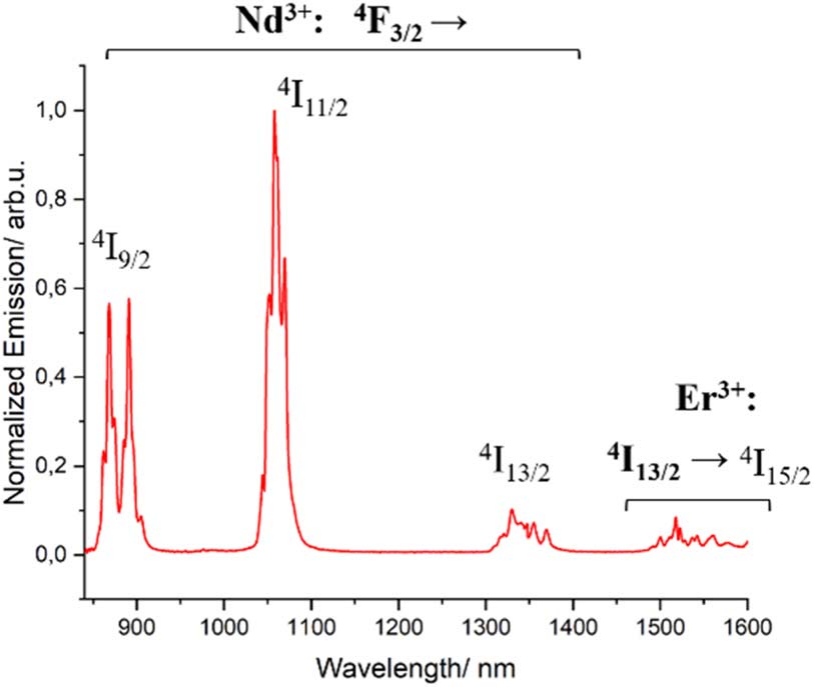
Emission (*λ*_exc_ = 400 nm, red trace) spectrum of complex 3 in MeOH-d_4_ : DMSO-d_6_ (1 : 1) at 77 K.

Complex 3 [ErNdEr] was then analyzed to investigate the possible photophysical intercommunication between the two different centers present within the same molecule. The emission spectra upon excitation of the singlet state of the ligands (*λ*_exc_ = 400 nm), both in solution and the solid state, show luminescence coming from both the ^4^F_3/2_ state of Nd^3+^ and the ^4^I_13/2_ one of Er^3+^ (Fig. 5 and S12†). However, this does not allow confirming the ET between both centers. It only demonstrates an efficient antenna effect occurring when the ligands of the mixed-metal complex are brought to their excited states.

**Fig. 6:**
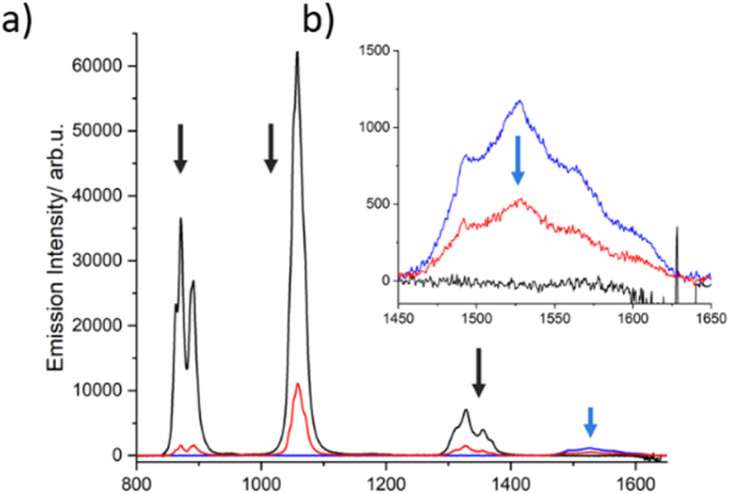
Emission spectra (*λ*_exc_ = 400 nm) of iso-absorbant solutions of complexes (1) (black trace), (2) (blue trace), and (3) (red trace) in MeOD-d_4_ : DMSO-d_6_ (1 : 1) solution at room temperature (a) with the corresponding Er^3+^ emission zoom (b).

To investigate a possible direct ET between both lanthanides, dilute solutions of complexes 1, 2 and 3 with identical optical density (OD = 0.4 at 400 nm, Fig. S13†) were compared at room temperature, which allowed evaluating changes in intensity of the emission bands. As illustrated in Fig. 6, the emission intensity of Nd^3+^ decreases significantly (∼80%) in going from complex 1 to 3. This pronounced decline cannot be solely attributed to the increase in the number of lanthanide acceptors (one Nd^3+^ in complex 1*vs.* two Er^3+^ and one Nd^3+^ in complex 3). The data therefore suggest that another deactivation pathway of Nd^3+^ may be occurring, which we anticipate could be a direct ET to Er^3+^. This, however, is not substantiated by a concomitant increase of the Er^3+^ emission in complex 3. In contrast, a clear decrease of this emission is observed in comparison to complex 2. The dilution effect is again evident when changing from complex 2 (two Er^3+^) to 3 (two Er^3+^ and one Nd^3+^), resulting in an emission decrease of 1/3. However, the reduction of the Er^3+^ emission (∼50%) is higher than expected, suggesting that a possible back ET to Nd^3+^ could be also occurring. The same effect was measured at 77 K. However, the decrease of the Nd^3+^ and Er^3+^ centered bands of complex 3 was less evident at low temperature (Fig. S14†). Therefore, further verification was required.

**Fig. 7:**
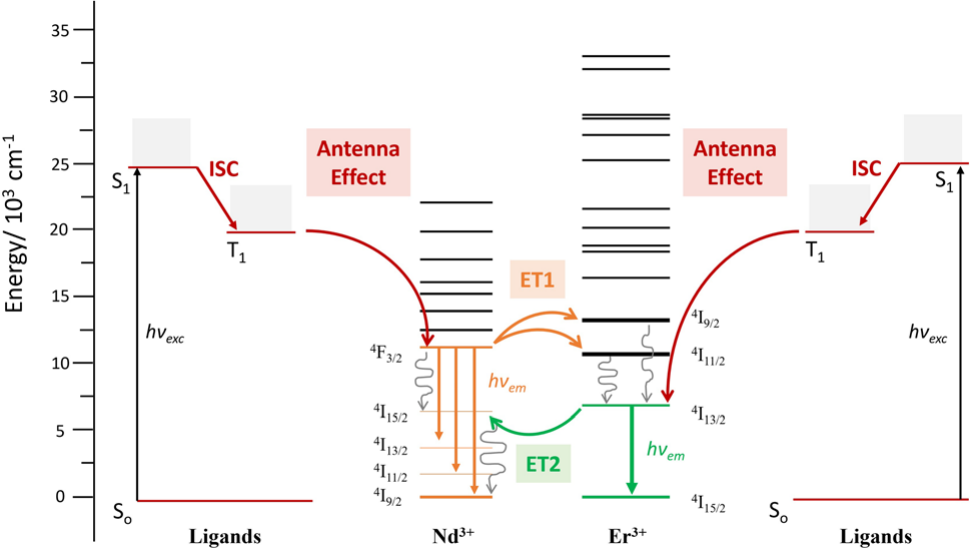
Energy diagram levels and terms involved in the ET phenomena occurring within complex 3 [ErNdEr].

Since the hypothesis of a back ET is unprecedented at the molecular level, a quantitative analysis was carried out. Thus, the lifetimes of complex 3 were determined in solution at 1058 nm and 1530 nm (Fig. S15†). The excited state decay at 1058 nm (^4^F_3/2_ state of Nd^3+^) was satisfactorily fitted to a monoexponential function with a lifetime of 1.00 μs at room temperature. A clear shortening of the lifetime values is observed in comparison with those obtained for the [LuNdLu] (1) complex (3.99 μs). Indeed, by using the following equation:2
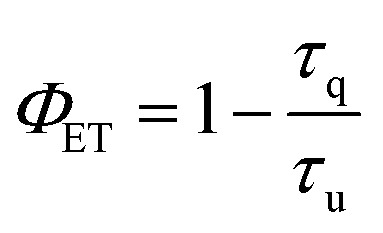


the reduction of the lifetime can be translated directly into the efficiency of the energy transfer, amounting to 75% at room temperature. This energy transfer (ET1) is expected to occur from the ^4^F_3/2_ state of Nd^3+^ to the approximately isoenergetic state/s of Er^3+^, *i.e.*^4^I_11/2_ and/or ^4^I_9/2_ (Fig. 7).^74^ A similar lifetime variation is observed at 77 K (Table 1) but the effect is less pronounced, leading to a ET1 efficiency of 51%. The fact that the ET1 was found to be lower at 77 K may reflect the fact that at low temperature the transfer ^4^F_3/2_ (Nd^3+^) → ^4^I_9/2_ (Er^3+^) is not efficient, since the latter (^4^I_9/2_ of Er^3+^) lies slightly higher in energy (13 089 cm^−1^) than the ^4^F_3/2_ state of Nd^3+^ (11 338 cm^−1^), as determined from the excitation spectra of “pure” complexes 1 and 2, respectively (see above). The possibility of the reverse effect, *i.e.* the energy transfer from Er^3+^ to Nd^3+^, was also investigated by lifetime determination. This was conducted by assessing the excited state decay at 1530 nm (that occurs from the state ^4^I_13/2_ of Er^3+^) in complex 3. The mono-exponential fitting of the decay gave lifetime values of 1.90 μs at room temperature, compared to 2.85 μs for 2 in the absence of Nd^3+^, suggesting an Er-to-Nd energy transfer. In this case, this remarkable transfer (ET2, Fig. 7) occurring from the ^4^I_13/2_ state of Er^3+^ may be taking place to the ^4^I_15/2_ or a lower lying state of Nd^3+^.^74,75^ In contrast to the first type of transfer (ET1), ET2 does not result in emission from Nd^3+^ but into non-radiative decay processes as the ultimate outcome of the partial deactivation of the Er^3+^ emission. Furthermore, this transfer was also unveiled at 77 K, where a lifetime of 2.3 μs was determined following a mono-exponential fitting, compared to 3.5 μs for the pure Er^3+^ complex.

**Fig. 8:**
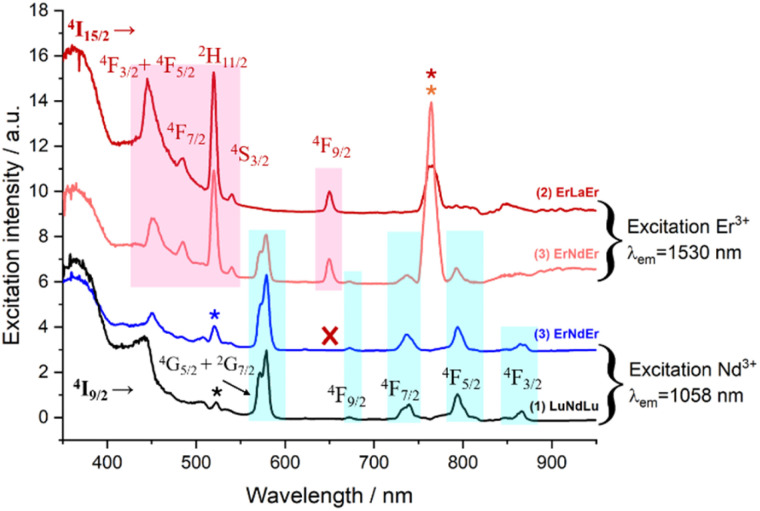
Excitation spectra of complexes (1) at *λ*_em_ = 1058 nm (black trace), (2) at *λ*_em_ = 1530 nm (red trace) and (3) at *λ*_em_ = 1058 nm (blue trace) and at *λ*_em_ = 1530 nm (orange trace) in MeOH-d_4_/DMSO-d_6_ (1 : 1) at 77 K.

Excitation spectra allowed further characterization of this rare intramolecular double ET phenomenon. These were obtained by generating f–f emission transitions within complex 3 in frozen solution. When fixing the detected emission wavelength at 1056 (Nd^3+^ emission), the main excitations lines could be assigned, as expected, to transitions from the ^4^I_9/2_ state of Nd^3+^ as for complex 1 (Fig. 8). On the other hand, no excitations of Er^3+^ were detected to generate any emission from Nd^3+^. This observation is not in contradiction with ET2 (Er-to-Nd) but rather corroborates it, as this type of transfer only results in non-radiative deactivation of Er^3+^ instead of any radiative emission (Fig. 8). On the other hand, when the emission was fixed at 1550 nm (Er^3+^ emission), two sets of lines could be identified; those from transitions arising from the ^4^I_15/2_ state of Er^3+^ (also seen in complex 2) but also, some from exciting the ^4^I_9/2_ state of Nd^3+^ (Fig. 8). This further confirms the direct ET1 (Nd-to-Er) discussed above.

## Conclusions

The pure heterometallic [ErNdEr] complex synthesized here allows unveiling the first instance of intramolecular ET between the Nd and Er centers being studied at the molecular level. The ideal distance between both ions (∼4 Å) and the specific molecular scaffold have been identified as key parameters in the analysis of this transfer. Indeed, the photophysical analysis reveals a rare intramolecular double ET phenomenon. A comparison of the excited state lifetime values in the presence and absence of the lanthanide quencher (Er^3+^ in the case of the Nd-to-Er or Nd^3+^ in the case of Er-to-Nd ET) indicated the presence of two distinct types of transfer. The first one (ET1) occurs from the ^4^F_3/2_ state of Nd^3+^ to the approximately isoenergetic states of Er^3+^, *i.e.*^4^I_11/2_ and/or ^4^I_9/2_, which results in the emission of the Er^3+ 4^I_11/2_ state at 1530 nm. In contrast, ET2 occurs from the ^4^I_13/2_ state of Er^3+^ to the ^4^I_15/2_ or lower-lying states of Nd^3+^ and does not result in Nd^3+^ emission but in non-radiative decay processes, as the ultimate outcome of the partial deactivation of the Er^3+^ emission. This unprecedented observation was first qualitatively detected by comparing the emission of iso-absorbant solutions and quantitatively confirmed by studying the excited state decays as well as by a critical excitation spectra analysis. The availability of a rich collection of pure heterometallic [LnLn′Ln] complexes offers a great opportunity to discover new intermetallic ET phenomena at the molecular level and their very precise analysis.

## Data availability

The data supporting this article, including crystallographic details, information on synthetic procedures, ESI-MS, absorption, emission, lifetime measurements and fits and NMR experiments, magnetic data treatment, and DFT calculations, have been included as part of the ESI.†

## Author contributions

DM, coordination chemistry synthesis and collection of photophysical data; AS, collection of photophysical data and interpretation; LAB, conceptualization, ligand synthesis and coordination chemistry synthesis; DA, conceptualization and coordination chemistry synthesis; OR, crystal structure determination and magnetic properties; YG, collection of photophysical data and interpretation; FR, conceptualization and photophysical data interpretation; OM, conceptualization and photophysical data interpretation; LAG, conceptualization, photophysical data interpretation and paper writing; GA, research coordination, conceptualization and paper writing.

## Conflicts of interest

There are no conflicts to declare.

## Supplementary Material

SC-OLF-D4SC03994G-s001

SC-OLF-D4SC03994G-s002

## References

[cit1] Xian T., Meng Q., Gao F., Hu M., Wang X. (2023). Coord. Chem. Rev..

[cit2] Bünzli J.-C. G. (2010). Chem. Rev..

[cit3] Heffern M. C., Matosziuk L. M., Meade T. J. (2014). Chem. Rev..

[cit4] Bünzli J.-C. G., Eliseeva S. V. (2010). J. Rare Earths.

[cit5] Tessitore G., Mandl G. A., Maurizio S. L., Kaur M., Capobianco J. A. (2023). RSC Adv..

[cit6] Monteiro J. H. S. K. (2020). Molecules.

[cit7] Eliseeva S. V., Bünzli J.-C. G. (2010). Chem. Soc. Rev..

[cit8] Bünzli J.-C. G. and Chauvin A.-S., in Handbook on the Physics and Chemistry of Rare Earths, ed. J.-C. G. Bünzli and V. K. Pecharsky, Elsevier, 2014, vol. 44, pp. 169–281

[cit9] Richards B. S., Hudry D., Busko D., Turshatov A., Howard I. A. (2021). Chem. Rev..

[cit10] Van Der Ende B. M., Aarts L., Meijerink A. (2009). Phys. Chem. Chem. Phys..

[cit11] Mackenzie L. E., Pal R. (2020). Nat. Rev. Chem.

[cit12] Bünzli J.-C. G. and Eliseeva S. V., Basics of Lanthanide Photophysics, Springer, Berlin Heidelberg, 2010, pp. 1–45, 10.1007/4243_2010_3

[cit13] D’Aléo A., Pointillart F., Ouahab L., Andraud C., Maury O. (2012). Coord. Chem. Rev..

[cit14] Pope S. J. A., Coe B. J., Faulkner S., Bichenkova E. V., Yu X., Douglas K. T. (2004). J. Am. Chem. Soc..

[cit15] Ward M. D. (2010). Coord. Chem. Rev..

[cit16] Xu L.-J., Xu G.-T., Chen Z.-N. (2014). Coord. Chem. Rev..

[cit17] Nadort A., Zhao J., Goldys E. M. (2016). Nanoscale.

[cit18] Zhao S., Yu D., Li B., Kanwal S., Shen T., Wu J., Zhuang S., Zhang D. (2024). Adv. Opt. Mater..

[cit19] Safdar M., Ghazy A., Lastusaari M., Karppinen M. (2020). J. Mater. Chem. C.

[cit20] David P. S., Panigrahi P., Raman S., Nagarajan G. S. (2021). Mater. Sci. Semicond. Process..

[cit21] Duan C., Liang L., Li L., Zhang R., Xu Z. P. (2018). J. Mater. Chem. B.

[cit22] Zhao H., Li Y., Zhang X., Wu K., Lv J., Chen C., Liu H., Shi Z., Ju H., Liu Y. (2022). Biomaterials.

[cit23] Mettenbrink E. M., Yang W., Wilhelm S. (2022). Adv. Photonics Res..

[cit24] Malhotra K., Hrovat D., Kumar B., Qu G., Houten J. V., Ahmed R., Piunno P. A. E., Gunning P. T., Krull U. J. (2023). ACS Appl. Mater. Interfaces.

[cit25] Charbonnière L. J., Nonat A. M., Knighton R. C., Godec L. (2024). Chem. Sci..

[cit26] Knighton R. C., Soro L. K., Francés-Soriano L., Rodríguez-Rodríguez A., Pilet G., Lenertz M., Platas-Iglesias C., Hildebrandt N., Charbonnière L. J. (2022). Angew. Chem., Int. Ed..

[cit27] Nonat A., Bahamyirou S., Lecointre A., Przybilla F., Mély Y., Platas-Iglesias C., Camerel F., Jeannin O., Charbonnière L. J. (2019). J. Am. Chem. Soc..

[cit28] Hernández I., Pathumakanthar N., Wyatt P. B., Gillin W. P. (2010). Adv. Mater..

[cit29] Balashova T. V., Pushkarev A. P., Yablonskiy A. N., Andreev B. A., Grishin I. D., Rumyantcev R. V., Fukin G. K., Bochkarev M. N. (2017). J. Lumin..

[cit30] Wang J., Jiang Y., Liu J.-Y., Xu H.-B., Zhang Y.-X., Peng X., Kurmoo M., Ng S. W., Zeng M.-H. (2021). Angew. Chem., Int. Ed..

[cit31] Gálico D. A., Ovens J. S., Sigoli F. A., Murugesu M. (2021). ACS Nano.

[cit32] Goderski S., Runowski M., Woźny P., Lavín V., Lis S. (2020). ACS Appl. Mater. Interfaces.

[cit33] Gupta A., Ghosh S., Thakur M. K., Zhou J., Ostrikov K., Jin D., Chattopadhyay S. (2021). Prog. Mater. Sci..

[cit34] Qin X., Xu J., Wu Y., Liu X. (2019). ACS Cent. Sci..

[cit35] Lis S., Elbanowski M., Mąkowska B., Hnatejko Z. (2002). J. Photochem. Photobiol., A.

[cit36] Lewis D. J., Glover P. B., Solomons M. C., Pikramenou Z. (2011). J. Am. Chem. Soc..

[cit37] Egger C., Guénée L., Deorukhkar N., Piguet C. (2024). Dalton Trans..

[cit38] Artizzu F., Quochi F., Marchiò L., Correia R. F., Saba M., Serpe A., Mura A., Mercuri M. L., Bongiovanni G., Deplano P. (2015). Chem.–Eur. J..

[cit39] André N., Jensen T. B., Scopelliti R., Imbert D., Elhabiri M., Hopfgartner G., Piguet C., Bünzli J.-C. G. (2004). Inorg. Chem..

[cit40] Floquet S., Borkovec M., Bernardinelli G., Pinto A., Leuthold L.-A., Hopfgartner G., Imbert D., Bünzli J.-C. G., Piguet C. (2004). Chem.–Eur. J..

[cit41] Artizzu F., Quochi F., Marchiò L., Saba M., Serpe A., Mura A., Mercuri M. L., Bongiovanni G., Deplano P. (2016). MRS Adv..

[cit42] Placidi M. P., Villaraza A. J. L., Natrajan L. S., Sykes D., Kenwright A. M., Faulkner S. (2009). J. Am. Chem. Soc..

[cit43] Sørensen T. J., Tropiano M., Blackburn O. A., Tilney J. A., Kenwright A. M., Faulkner S. (2013). Chem. Commun..

[cit44] Le Roy J. J., Cremers J., Thomlinson I. A., Slota M., Myers W. K., Horton P. H., Coles S. J., Anderson H. L., Bogani L. (2018). Chem. Sci..

[cit45] Buch C. D., Hansen S. H., Mitcov D., Tram C. M., Nichol G. S., Brechin E. K., Piligkos S. (2021). Chem. Sci..

[cit46] Aguilà D., Barrios L. A., Velasco V., Roubeau O., Repollés A., Alonso P. J., Sesé J., Teat S. J., Luis F., Aromí G. (2014). J. Am. Chem. Soc..

[cit47] González-Fabra J., Bandeira N. A. G., Velasco V., Barrios L. A., Aguila D., Teat S. J., Roubeau O., Bo C., Aromí G. (2017). Chem.–Eur. J..

[cit48] Aguilà D., Velasco V., Barrios L. A., González-Fabra J., Bo C., Teat S. J., Roubeau O., Aromí G. (2018). Inorg. Chem..

[cit49] Velasco V., Barrios L. A., Schütze M., Roubeau O., Luis F., Teat S. J., Aguilà D., Aromí G. (2019). Chem.–Eur. J..

[cit50] Abad Galán L., Aguilà D., Guyot Y., Velasco V., Roubeau O., Teat S. J., Massi M., Aromí G. (2021). Chem.–Eur. J..

[cit51] Maniaki D., Sickinger A., Barrios L. A., Aguilà D., Roubeau O., Settineri N. S., Guyot Y., Riobé F., Maury O., Galán L. A., Aromí G. (2023). Inorg. Chem..

[cit52] Xu H.-B., Deng J.-G., Zhang L.-Y., Chen Z.-N. (2013). Cryst. Growth Des..

[cit53] Yao H., Calvez G., Daiguebonne C., Bernot K., Suffren Y., Puget M., Lescop C., Guillou O. (2017). Inorg. Chem..

[cit54] Chuasaard T., Jittipiboonwat P., Ngamjarurojana A., Yotnoi B., Rujiwatra A. (2023). J. Solid State Chem..

[cit55] Abad Galán L., Sobolev A. N., Skelton B. W., Zysman-Colman E., Ogden M. I., Massi M. (2018). Dalton Trans..

[cit56] Artizzu F., Serpe A., Marchiò L., Saba M., Mura A., Mercuri M. L., Bongiovanni G., Deplano P., Quochi F. (2015). J. Mater. Chem. C.

[cit57] Oggianu M., Mameli V., Hernández-Rodríguez M. A., Monni N., Souto M., Brites C. D. S., Cannas C., Manna F., Quochi F., Cadoni E., Masciocchi N., Carneiro Neto A. N., Carlos L. D., Mercuri M. L. (2024). Chem. Mater..

[cit58] Faulkner S., Pope S. J. A. (2003). J. Am. Chem. Soc..

[cit59] Artizzu F., Quochi F., Marchiò L., Sessini E., Saba M., Serpe A., Mura A., Mercuri M. L., Bongiovanni G., Deplano P. (2013). J. Phys. Chem. Lett..

[cit60] Artizzu F., Quochi F., Marchiò L., Figus C., Loche D., Atzori M., Sarritzu V., Kaczmarek A. M., Van Deun R., Saba M., Serpe A., Mura A., Mercuri M. L., Bongiovanni G., Deplano P. (2015). Chem. Mater..

[cit61] Song L., Wang Q., Tang D., Liu X., Zhen Z. (2007). New J. Chem..

[cit62] Shi W. Q., Bass M., Birnbaum M. (1989). J. Opt. Soc. Am. B.

[cit63] Barbosa-García O., McFarlane R. A., Birnbaum M., Díaz-Torres L. A. (1997). J. Opt. Soc. Am. B.

[cit64] Macaluso E., Rubín M., Aguilà D., Chiesa A., Barrios L. A., Martínez J. I., Alonso P. J., Roubeau O., Luis F., Aromí G., Carretta S. (2020). Chem. Sci..

[cit65] Forgan R. S. (2020). Chem. Sci..

[cit66] Alemany P., Casanova D., Alvarez S., Dryzun C., Avnir D. (2017). Rev. Comput. Chem..

[cit67] Bettencourt-Dias A. d. , Luminescence of lanthanide ions in coordination compounds and nanomaterials, Wiley, Chichester, U.K., 2014

[cit68] Tan R. H. C., Motevalli M., Abrahams I., Wyatt P. B., Gillin W. P. (2006). J. Phys. Chem. B.

[cit69] Galán L. A., Reid B. L., Stagni S., Sobolev A. N., Skelton B. W., Cocchi M., Malicka J. M., Zysman-Colman E., Moore E. G., Ogden M. I., Massi M. (2017). Inorg. Chem..

[cit70] Martín-Ramos P., Ramos Silva M., Lahoz F., Martín I. R., Chamorro-Posada P., Eusebio M. E. S., Lavín V., Martín-Gil J. (2014). J. Photochem. Photobiol., A.

[cit71] Sickinger A., Baguenard B., Bensalah-Ledoux A., Guyot Y., Guy L., Pointillart F., Cador O., Grasser M., Le Guennic B., Riobé F., Maury O., Guy S. (2024). J. Mater. Chem. C.

[cit72] Martín-Ramos P., Martín I. R., Lahoz F., Hernández-Navarro S., Pereira da Silva P. S., Hernández I., Lavín V., Ramos Silva M. (2015). J. Alloys Compd..

[cit73] Ahmed Z., Aderne R. E., Kai J., Resende J. A. L. C., Padilla-Chavarría H. I., Cremona M. (2017). RSC Adv..

[cit74] Liu Y., Sun Y., Wang Y., You Z., Zhu Z., Li J., Tu C. (2018). J. Lumin..

[cit75] Wang Y., Li J., Zhu Z., You Z., Xu J., Tu C. (2015). Opt. Express.

