# Supplementary material for: Energy exchange between Nd3+ and Er3+ centers within molecular complexes
Source: Chem Sci. 2024 Oct 18. Online ahead of print. doi: 10.1039/d4sc03994g (PMC11515939; doi:10.1039/d4sc03994g)
Supplement: SC-OLF-D4SC03994G-s001 [file SC-OLF-D4SC03994G-s001.pdf]

Supplementary Information (SI) for Chemical Science.
This journal is © The Royal Society of Chemistry 2024

Energy exchange between Nd^{3+} and Er^{3+} centers within molecular complexes

Diamantoula Maniaki,[a,b] Annika Sickinger,[d] Leoní A. Barrios,[a,b] David Aguilà,[a,b] Olivier Roubeau,[e] Yannick Guyot,[h] François Riobé,[d] Olivier Maury[d] Laura Abad Galán,[c,*] and Guillem Aromí [a,b,*]

SUPPORTING INFORMATION

Synthesis

Ligand H_2LA was prepared according to our published procedure.[1] Ligand H_2LB was prepared as previously reported by our group.[2] Complexes $[Lu_2Nd(LA)_2(LB)_2(py)(H_2O)_2](NO_3)$ (**1**)[3] and $[Er_2La(LA)_2(LB)_2(py)(H_2O)_2](NO_3)$ (**2**)[4] were prepared according to procedures previously published by our group.

$[Er_2Nd(LA)_2(LB)_2(py)(H_2O)_2](NO_3)$ (3). A yellow solution of H_2LA (14.8 mg, 0.032 mmol) and H_2LB (10.2 mg, 0.032 mmol) in pyridine (10 mL) was added dropwise under stirring to a light blue solution of $Nd(NO_3)_3{\cdot}6H_2O$ (7 mg, 0.016 mmol), $Er(NO_3)_3{\cdot}5H_2O$ (14.2 mg, 0.032 mmol) and $CuCl_2{\cdot}2H_2O$ (2.7 mg, 0.016 mmol) in pyridine (10 mL). The resulting green solution was left under stirring for 1 h and layered with hexane. After a week, green crystals of $[Cu(py)_4(NO_3)_2]$ were formed. After one month, large orange crystals also formed, which were easily separated from the rest. Yield: ~15%. Elemental analysis (C,H,N): calcd (found) for **3**$\cdot 2.6H_2O$: C, 55.4 (55.18); H 3.29 (3.05); N. 3.69 (4.03). Metal analysis (Nd/Er molar ratio, ICP): calc 0.50 (0.53). MS: m/z = 2052.1507 $[NdEr_2(LA)_2(LB)_2]^+$, 1026.0958 $([NdEr_2(LA)_2(LB)_2]+H^+)^{2+}$, 1037.5852 $([NdEr_2(LA)_2(LB)_2]+Na^+)^{2+}$.

Other Physical Measurements

Elemental analyses were performed with a Perkin-Elmer Series II CHNS/O Analyzer 2400 (C, H, N) at the Servei de Microanàlisi of CSIC, Barcelona. IR spectra were recorded as KBr pellet samples on a Nicolet 5700 FTIR spectrometer. Inductively coupled plasma atomic emission spectroscopy (ICP-AES) experiments were performed by using a Perkin Elmer Elan 6000, at the CCiTUB; 5mg of crystals of **3** and 1 mL of HNO_3 were added to UltraWave tubes, and heated to 240º. Ultrapure H_2O (20 Ml) was then added. After homogenization, the solution was transferred to 14mL plastic tubes. The weight/volume ratio results as % results, gave metal molar ratios of 1 Nd : 2 Er.

---

[1] Barrios L. A., Peyrecave-Lleixa E., Craig G. A., Roubeau O., Teat S. J., Aromí G., *Eur. J. Inorg. Chem.*, **2014**, 6013-6021.
[2] Velasco V., Barrios L. A., Schütze M., Roubeau O., Luis F., Teat S. J., Aguilà D., Aromí G., *Chem., Eur. J.*, **2019**, *25*, 15228 – 15232.
[3] D. Maniaki, A. Sickinger, L. A. B. Moreno, D. Aguila, O. Roubeau, N. S. Settineri, Y. Guyot, F. Riobe, O. Maury, L. A. Galan and G. Aromi, *Inorg. Chem.*, 2023, **62**, 3106-3115.
[4] E. Macaluso, M. Rubin, D. Aguila, A. Chiesa, L. A. Barrios, J. I. Martinez, P. J. Alonso, O. Roubeau, F. Luis, G. Aromi and S. Carretta, *Chem. Sci.*, 2020, **11**, 10337-10343.

Magnetic properties.

The temperature dependence of the magnetic susceptibility χ in the range 2-295 K under an applied field of 0.1 T and the isothermal variable field magnetization at 2 and 5 K were determined for a bulk polycrystalline sample of **3** through *dc* magnetization measurements performed with a commercial MPMS-XL SQUID magnetometer hosted at the Physical Measurements Unit of the Servicio General de Apoyo a la Investigación-SAI, Universidad de Zaragoza. Data were corrected for the contribution of the gelatine sample capsule, determined empirically. Elemental analysis shows that compound **3** loses its lattice pyridine to absorb water from air, so that we have used the formula **3**·6py·5H_2O (FW = 2794.1 g/mol), considering that the more disordered lattice pyridines would be exchanged by air moist during the polycrystalline sample preparation.

The χT *vs.* T plot (Fig. S1) shows a value of χT at 295 K of 24.223 cm^3Kmol^{-1}, very close to the value of 24.60 cm^3Kmol^{-1} expected for two Er(III) ($^4I_{15/2}$; g_J = 6/5, J = 15/2) and one Nd(III) ion ($^4I_{9/2}$; g_J = 8/11, J = 9/2). The decline observed with decreasing temperature, down to 15.991 cm^3Kmol^{-1} at 2 K, can reasonably be attributed to depopulation of the Kramers doublets embedded within the J = 15/2 and J = 9/2 states, respectively. The experimental χT *vs.* T plot is indeed well-simulated by the sum of a simplified van Vleck susceptibility model[5] for two Er(III) and one Nd(III) ions, indicating that if a weak antiferromagnetic interaction is present, it is likely very weak. M *vs.* H data (inset in Fig. S1) at 2 and 5 K exhibit abrupt increases with increasing field levelling off at 5 T at 12.740 and 12.024 μ_B, respectively, although without reaching saturation, a likely result of the population of excited states.

[5] E. Bartolomé, P. J. Alonso, A. Arauzo, J. Luzón, J. Bartolomé, C. Racles and C. Turta, *Dalton Trans.*, 2012, **41**, 10382–10389

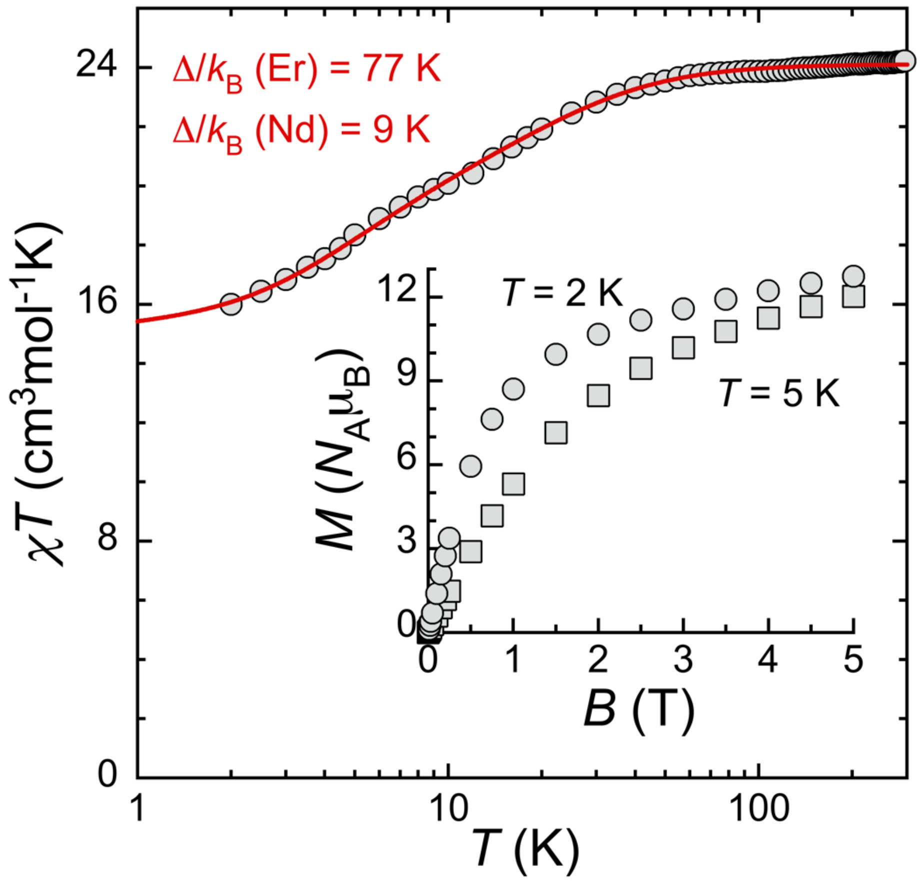

Figure S1. Temperature dependence of the magnetic susceptibility χ of **3**, plotted as χT *vs.* T. The full red line is the best fit obtained considering the sum of a simplified van Vleck susceptibility for one Nd(III) and two Er(III) with the indicated energy gaps Δ/k_B. The Er(III) ions parameters have been fixed as previously reported for the [ErLnEr] (Ln = La, Ce and Pr) analogues.[6] Inset: isothermal magnetization *vs.* field data at 2 and 5 K.

Mass Spectrometry

Positive-ion ESI mass spectrometry experiments were performed by using a LC/MSD-TOF (Agilent Technologies) with a dual source equipped with a lock spray for internal reference introduction, at the Unitat d'Espectrometria de Masses from the Universitat de Barcelona. Experimental parameters: capillary voltage 4 kV, gas temperature 325°C, nebulizing gas pressure 103.42 kPa, drying gas flow 7.0 L min-1 and fragmentor voltage 175- 250 V. Internal reference masses were m/z 121.05087 (purine) or 922.00979 (HP-0921). Crystals of **1**, **2** or **3** were dissolved in mixtures of MeOH with the minimal amount of DMSO and introduced into the source by using a HPLC system (Agilent 110) with a mixture of H_2O/CH_3CN (1:1) as the eluent (200 μL min^{-1}). As observed previously for

---

[6] a) E. Macaluso, M. Rubín, D. Aguilà, A. Chiesa, L. A. Barrios, J. I. Martínez, P. J. Alonso, O. Roubeau, F. Luis, G. Aromí and S. Carretta, *Chem. Sci.*, 2020, **11**, 10337–10343; b) D. Maniaki, D. Garay-Ruiz, L. A. Barrios, D. O. T. A. Martins, D. Aguilà, F. Tuna, D. Reta, O. Roubeau, C. Bo and G. Aromí, *Chem. Sci.*, 2022, **13**, 5574–5581

related clusters, the ionization caused the removal of both pyridine and water ligands from the complexes. For each complex, moieties related exclusively to the expected [LnLn'Ln] metal distribution were observed. Moreover, no signals for other metallic compositions were detected, thus evidencing not only the realization of the trinuclear heterometallic compound but also its robustness and exclusiveness in solution.

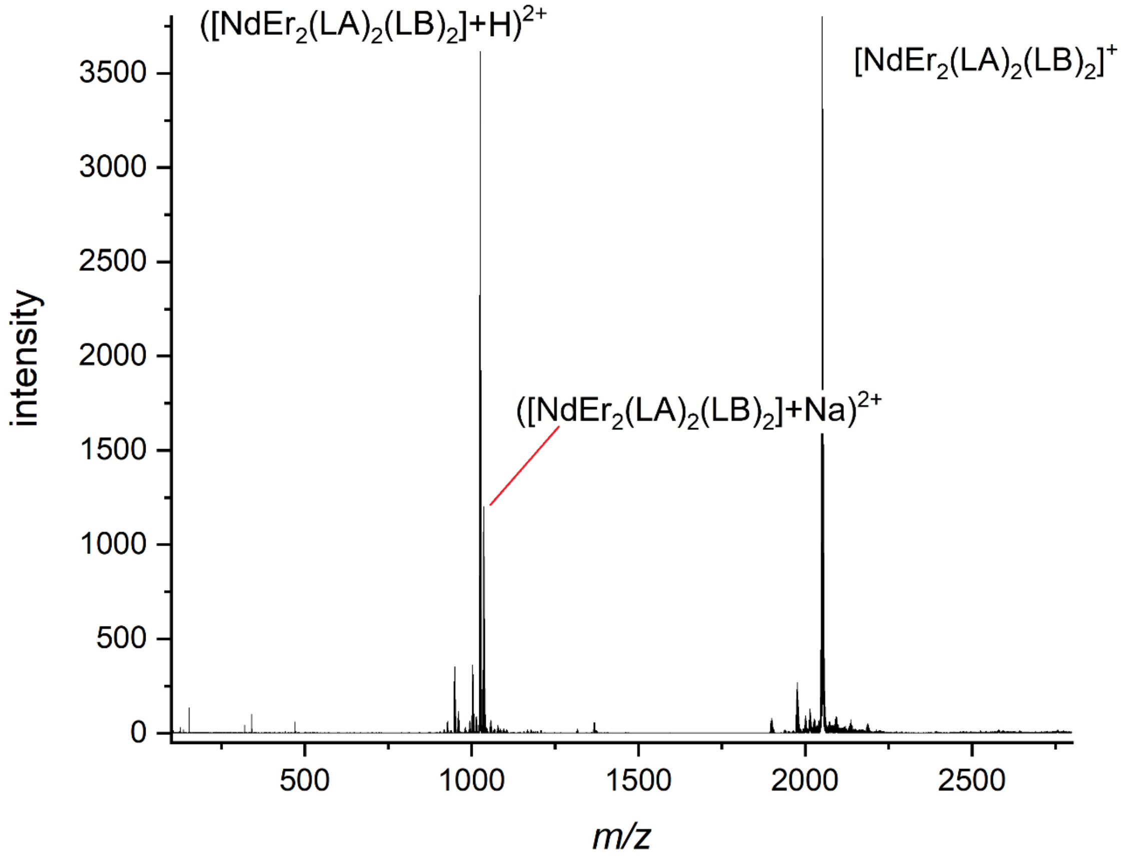

Figure S2. Full experimental ESI-MS spectrogram of compound **3** ([ErNdEr]), indicating the identity of the species producing the three main peaks of the diagram; $[Er_2Nd(LA)_2(LB)_2]^+$ (*m/z* = 2052.1507), $[Er_2Nd(LA)_2(LB)_2]+H)^{2+}$ (*m/z* = 1026.0958) and $[Er_2Nd(LA)_2(LB)_2]+Na)^{2+}$ (*m/z* = 1037.5852).

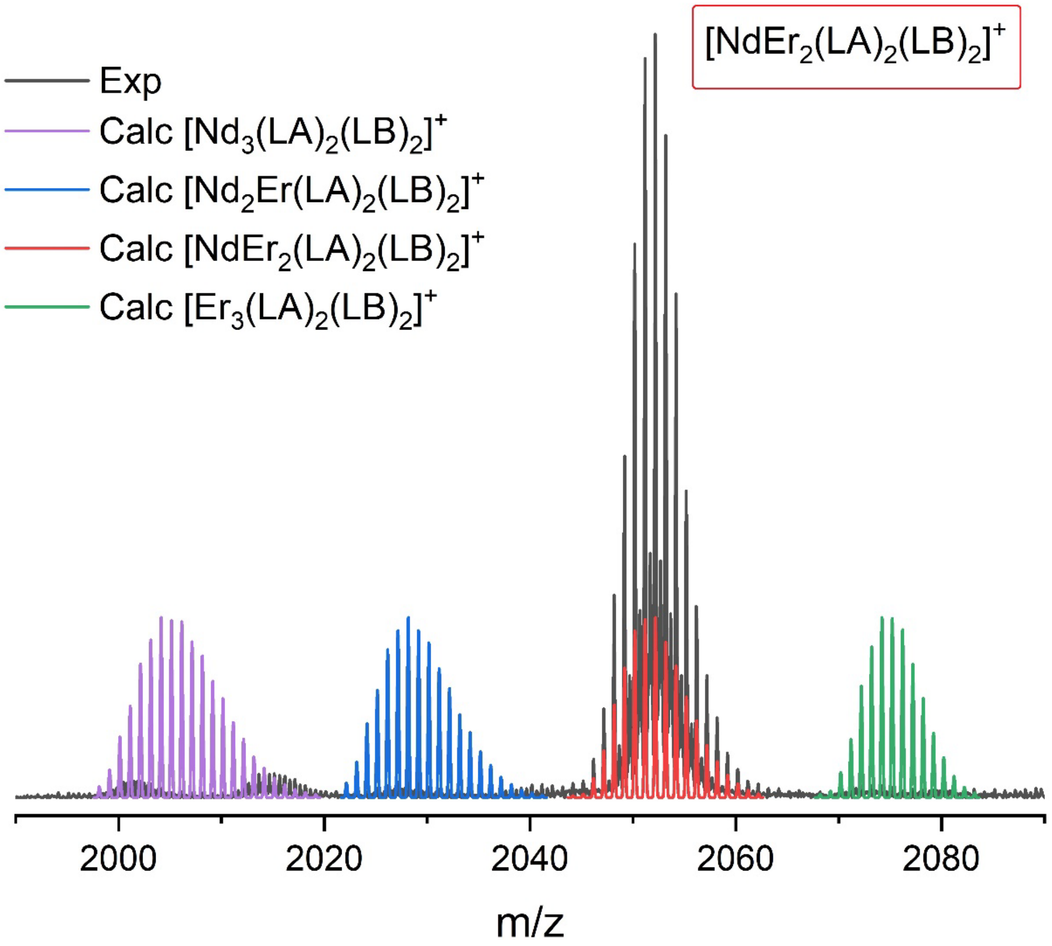

Figure S3. Selected region of the experimental (black line) ESI-MS spectrogram of compound **3** ([ErNdEr]), emphasizing the $[Er_2Nd(LA)_2(LB)_2]^+$ (*m/z* = 2052.1507) fragment, together with the corresponding calculated signals for the $[Nd_3]$ (purple line), $[ErNd_2]$ (blue line), $[Er_2Nd]$ (red line) and $[Er_3]$ (green line), metal distributions.

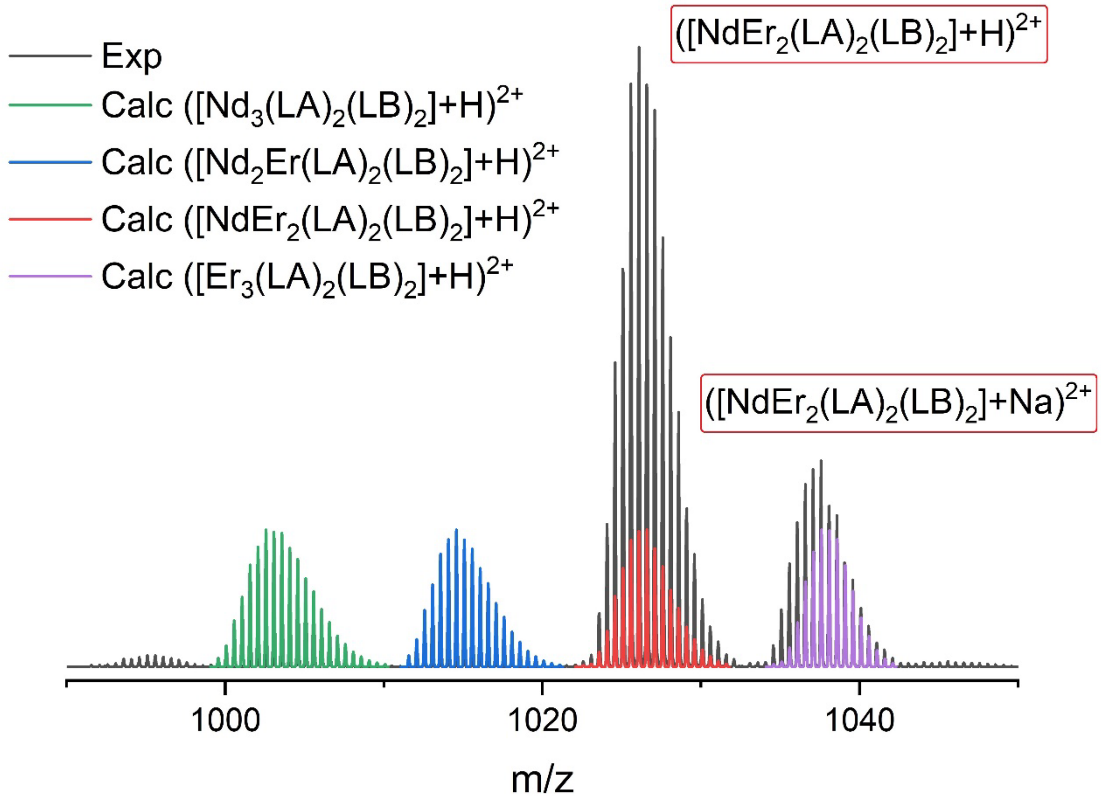

Figure S4. Selected region of the experimental (black line) ESI-MS spectrogram of compound **3** ([ErNdEr]), emphasizing the ($[Er_2Nd(LA)_2(LB)_2]+H)^{2+}$ (*m/z* = 1026.0958) fragment, together with the corresponding calculated signals for the [Nd_3] (green line), [$ErNd_2$] (blue line), [Er_2Nd] (red line) and [Er_3] (purple line), metal distributions. The experimental close lying signal from the fragment ($[NdEr_2(LA)_2(LB)_2]+Na^+)^{2+}$ (*m/z* = 1037.5852) is also indicated.

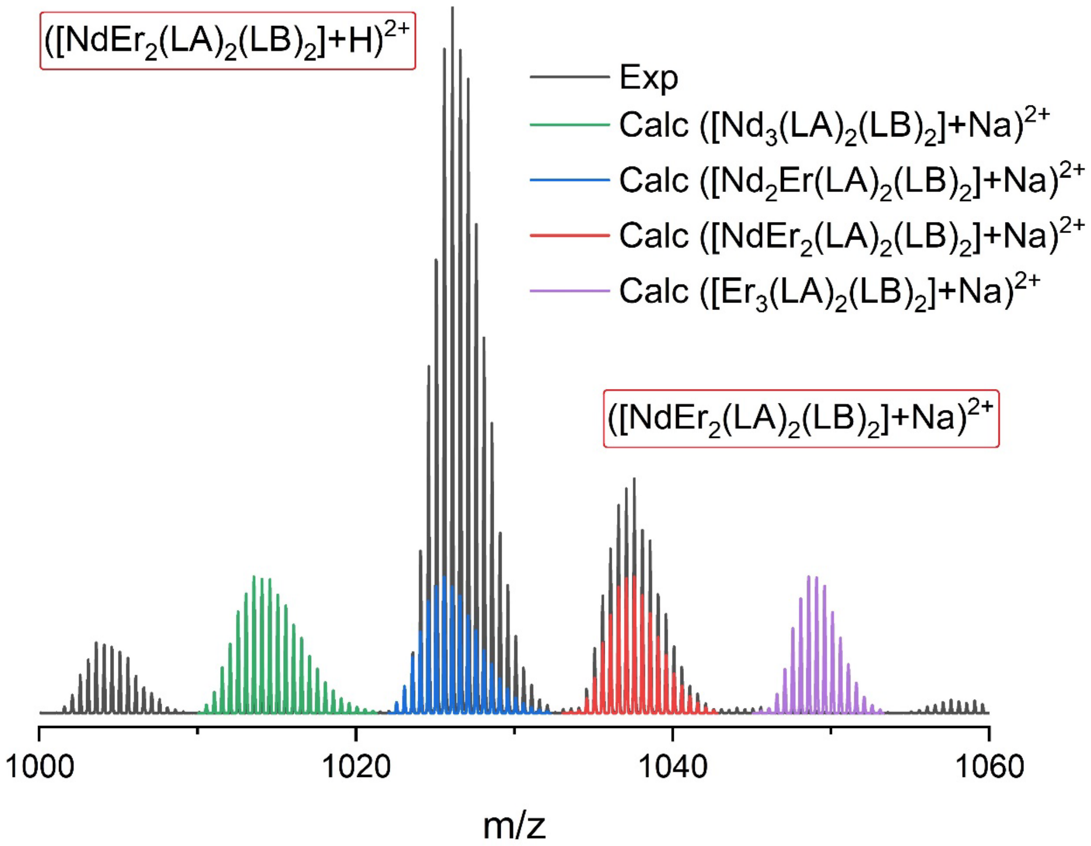

Figure S5. Selected region of the experimental (black line) ESI-MS spectrogram of compound **3** ([ErNdEr]), emphasizing the ($[Er_2Nd(LA)_2(LB)_2]+Na)^{2+}$ (*m/z* = 1037.5852) fragment, together with the corresponding calculated signals for the $[Nd_3]$ (green line), $[ErNd_2]$ (blue line), $[Er_2Nd]$ (red line) and $[Er_3]$ (purple line), metal distributions. The experimental close lying signal from the fragment ($[Er_2Nd(LA)_2(LB)_2]+H)^{2+}$ (*m/z* = 1026.0958) is also indicated.

Single Crystal X-ray Diffraction (SCXRD).

Data for compound **3** were obtained at 100 K on a yellow plate of dimensions 0.150 x 0.100 x 0.080 mm^3 using a Bruker Smart APEX diffractometer. Data reduction and absorption corrections were performed with SAINT and SADABS, respectively.[7] The structure was solved by intrinsic phasing with SHELXT[8] and refined by full-matrix least-squares on F^2 with SHELXL.[9]

All details can be found in CCDC 2351760. These data can be obtained free of charge from The Cambridge Crystallographic Data Center via https://summary.ccdc.cam.ac.uk/structure-summary-form. Crystallographic and refinement parameters are summarized in Table S1. Ln–ligand bond lengths and angles and Ln···Ln separations and hydrogen bonds details are given in Tables S2 and S3.

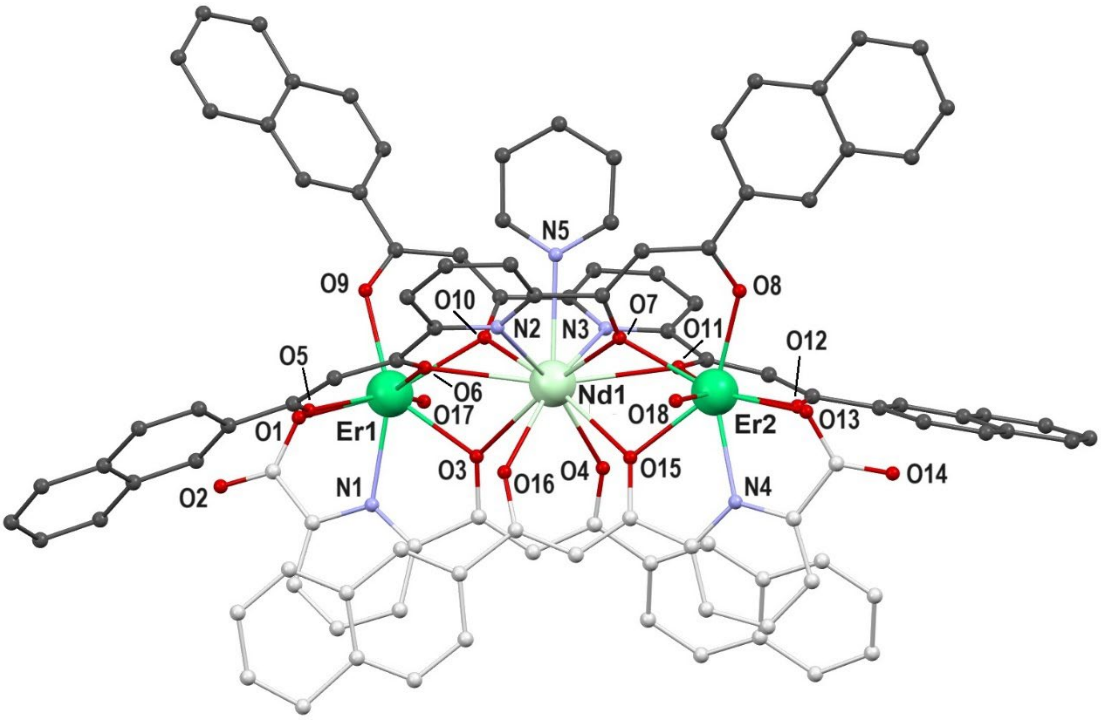

Figure S6. View of the cation $[Er_2Nd(LA)_2(LB)_2(py)(H_2O)_2]^+$ of **3** with heteroatoms labelled (C atoms in grey). H atoms not shown.

---

[7] G. M. Sheldrick, *SAINT and SADABS*, Bruker AXS Inc., Madison, Wisconsin, USA.
[8] G. M. Sheldrick, *Acta Cryst. A*, 2015, **71**, 3–8
[9] G. M. Sheldrick, *Acta Cryst. C*, 2015, **71**, 3–8

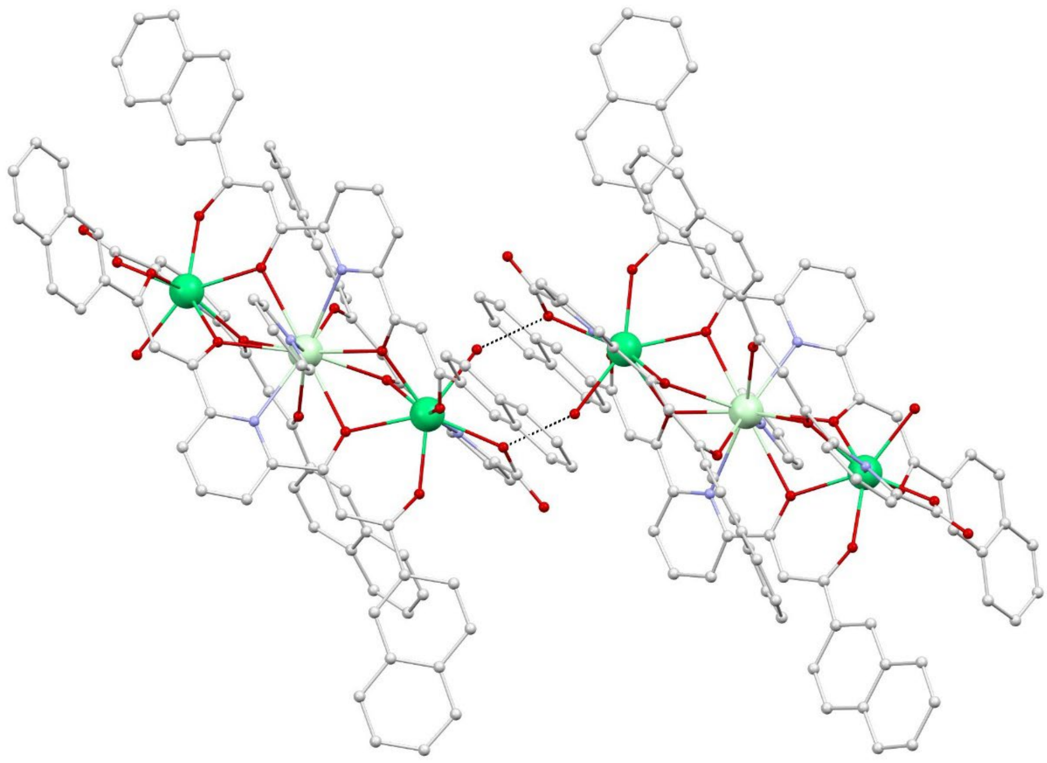

Figure S7. View of two cations of **3** highlighting hydrogen bonding interactions between them (dashed lines, see Table S3 for details).

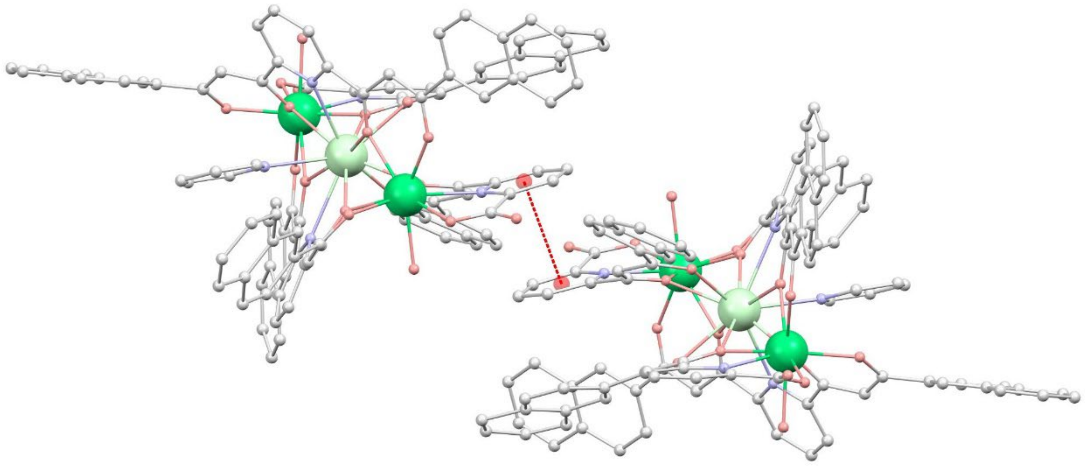

Figure S8. View of two cations of **3** highlighting a π···π interaction between two pyridyl groups from LB^{2-} ligands (red dashed lines, inter-centroid distance, 3.349 Å), with centroids shown as red balls.

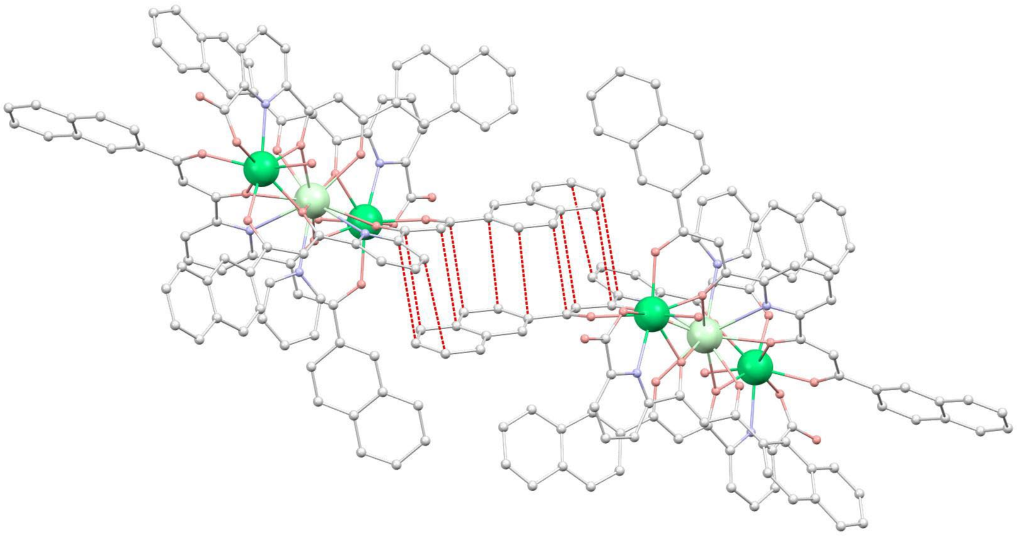

Figure S9. View of two cations of **3** highlighting seven π···π contacts between sp^2 C-atoms from two LA^{2-} ligands (red dashed lines, C···C distances ranging 3.377 to 3.854 Å).

Table S1. Crystallographic and refinement parameters for the structure of compound **3**.

	**3**
Formula	$C_{105}H_{69}Er_2N_5NdO_{18}$, NO_3, 11(C_5H_5N)
FW (g mol^{-1})	3099.51
T (K)	100
Wavelength (Å)	0.71093
Crystal system	triclinic
Space group	*P*–1
a (Å)	16.4316(16)
b (Å)	19.5670(17)
c (Å)	23.352(2)
α (°)	108.849(5)
β (°)	108.693(5)
γ (°)	92.157(5)
V (Å^3)	6646.6(11)
Z	2
ρ_{calcd} (g cm^{-3})	1.549
μ (mm^{-1})	1.716
Independent reflections (R_{int})	22606 (0.0853)
parameters / restraints	1909 / 1019
Goodness-of-fit	1.026
Final R1 / wR2 [I>2σ(I)]	0.0558 / 0.1320
Final R1 / wR2 [all data]	0.0833 / 0.1499
largest diff. peak / hole (e Å^3)	1.555 / –2.466

Table S2. Ln–ligand donor bond lengths (Å), Ln–O–Ln angles (º) and Ln···Ln separations (Å) in the structure of compound **3**.

Er1–O5	2.257(5)	Er1–O3–Nd1	105.50(17)
Er1–O9	2.290(5)	Er1–O6–Nd1	101.38(17)
Er1–O17	2.309(5)	Er1–O10–Nd1	107.24(16)
Er1–O3	2.325(5)		
Er1–O10	2.326(4)	Er2–O7–Nd1	107.85(19)
Er1–O1	2.347(5)	Er2–O11–Nd1	101.88(15)
Er1–O6	2.361(5)	Er2–O15–Nd1	104.06(16)
Er1–N1	2.388(6)		
Er2–O8	2.257(5)	Er1···Nd1	3.9438(6)
Er2–O12	2.278(5)	Er2···Nd1	3.9468(6)
Er2–O15	2.313(5)	Er1···Er2	7.881(1)
Er2–O7	2.319(5)	Er2···Er2[a]	6.059(1)
Er2–O18	2.335(5)		
Er2–O13	2.362(5)		
Er2–N4	2.384(6)		
Er2–O11	2.390(4)		
Nd1–O4	2.429(5)		
Nd1–O16	2.480(5)		
Nd1–O7	2.561(5)		
Nd1–O10	2.570(4)		
Nd1–O3	2.624(5)		
Nd1–O15	2.685(5)		
Nd1–O11	2.687(4)		
Nd1–N5	2.717(6)		
Nd1–O6	2.727(5)		
Nd1–N3	2.757(5)		
Nd1–N2	2.761(6)		

[a] -x, 1-y, 1-z

Table S3. Hydrogen bonds in the structure of compound **3**.

D–H···A	D–H (Å)	H···A (Å)	D···A (Å)	D–H···A (º)
O17–H17B···N1S	0.87	1.87	2.728(8)	168.3
O17–H17A···N2S	0.87	1.89	2.727(10)	161.2
O17–H17A···N12S	0.87	1.83	2.67(3)	160.3
O18–H18B···N3S	0.87	1.88	2.739(8)	171.0
O18–H18A···O13[a]	0.87	1.86	2.711(7)	165.4

[a] -x, 1-y, 1-z

Table S4. Continuous symmetry measures made on the coordination polyhedrons of the metals in complex **3**, using the program SHAPE for many of the possible ideal figures of each coordination number.

**Nd (CN = 8)**		
1. HP-11	D11h	Hendecagon
2. DPY-11	C10v	Decagonal pyramid
3. EBPY-11	D9h	Enneagonal bipyramid
4. JCPPR-11	C5v	Capped pentagonal prism J9
**5. JCPAPR-11**	**C5v**	**Capped pentagonal antiprism J11**
6. JAPPR-11	C2v	Augmented pentagonal prism J52
7. JASPC-11	Cs	Augmented sphenocorona J87

HP-11	DPY-11	EBPY-11	JCPPR-11	JCPAPR-11	JAPPR-11	JASPC-11
35.652	25.591	19.339	7.488	6.267	11.765	8.049

**Er (CN = 11)**		
1. OP-8	D8h	Octagon
2. HPY-8	C7v	Heptagonal pyramid
3. HBPY-8	D6h	Hexagonal bipyramid
4. CU-8	Oh	Cube
5. SAPR-8	D4d	Square antiprism
6. TDD-8	D2d	Triangular dodecahedron
7. JGBF-8	D2d	Johnson gyrobifastigium J26
8. JETBPY-8	D3h	Johnson elongated triangular bipyramid J14
9. JBTPR-8	C2v	Biaugmented trigonal prism J50
10. **BTPR-8**	**C2v**	**Biaugmented trigonal prism**
11. JSD-8	D2d	Snub diphenoid J84
12. TT-8	Td	Triakis tetrahedron

Er(1)											
OP-8	HPY-8	HBPY-8	CU-8	SAPR-8	TDD-8	JGBF-8	JETBPY-8	JBTPR-8	BTPR-8	JSD-8	TT-8
32.025	22.594	16.358	13.236	3.592	2.398	13.791	25.340	2.379	1.639	4.154	14.035
Er (2)											
OP-8	HPY-8	HBPY-8	CU-8	SAPR-8	TDD-8	JGBF-8	JETBPY-8	JBTPR-8	BTPR-8	JSD-8	TT-8
31.591	23.238	15.377	12.808	3.434	2.425	12.696	26.323	2.251	1.487	3.979	13.506

Photophysics

Absorption spectra were recorded on a JASCO V-650 spectrophotometer using complex solutions ($c \approx 10^{-5}$ M for absorption in the UV/vis region, optical density < 1) in deuterated DMSO-MeOH mixture (v/v = 1/1) using 10x10 mm quartz glass cuvettes. In this study we used iso-absorbant solutions at an optical density (OD) of 0.27. This value has been chosen because complexes **1**, **2** and **3** present stable and reproducible photophysical data over a period of several weeks. Iso-absorbant solutions have been used to compare the emission intensity among them and avoid the determination of luminescence quantum yields, keeping all experimental parameters identical (excitation and emission slits, excitation wavelength, detector and grating, integration times). The iso-absorbant solutions have been prepared by simply adjusting the OD to an identical value (here OD = 0.4) by careful dilution of a mother solution of the different compounds in the mixture of deuterated MeOH and DMSO (1:1).

The luminescence spectra were measured using a Horiba-Jobin Yvon Fluorolog-3® spectrofluorometer, equipped with a three slit double grating excitation monochromator with dispersions of 2.1 nm/mm (1200 grooves/mm) and a single grating iHR320 monochromator for the emission with a dispersion of 20 nm/mm (150 grooves/mm). The steady-state luminescence was excited by unpolarized light from a 450 W xenon CW lamp and detected at an angle of 90° through a FGL850 filter by a liquid nitrogen cooled Symphony® II CCD detector. Spectra were reference corrected for both the excitation source light intensity variation (lamp and grating) and the emission spectral response (detector and grating). For solid state and low temperature, the luminescence measurements were conducted on EPR glass tubes containing the complex either as solid powder or in solution. Otherwise, measurements were conducted on 10 x 10 mm cuvettes containing solutions at room temperature. For studies at 77K the samples were cooled by immersion of the EPR tube in liquid nitrogen using a transparent Dewar-type glass tube. It is important to note that the DMSO-MeOH mixture (v/v = 1:1) forms a transparent, isotropic organic glass at 77K enabling homogeneous irradiation and quantitative measurements.

NIR-luminescence lifetimes were determined using a home-made set-up for solution or solid-state samples in classical cuvettes or in EPR tubes (compatible with 77K measurements). The samples were excited using a pulsed Nd:YAG laser (SpectraPhysics) operating at 10 Hz. Light emitted at right angles to the excitation beam was focused onto the slits of a monochromator (PTI120), which was used to select the appropriate wavelength. The growth and decay of the luminescence at selected wavelengths was detected using a Ge photodiode (Edinburgh Instruments, EI-P) and recorded using a digital oscilloscope (Tektronix TDS320) before being transferred for analysis. Luminescence lifetimes were obtained by iterative reconvolution of the detector response (obtained by using a scatterer) with exponential components for growth and decay of the

metal-centered luminescence. Experimental uncertainties are estimated to be ±8% for this lifetime determination.

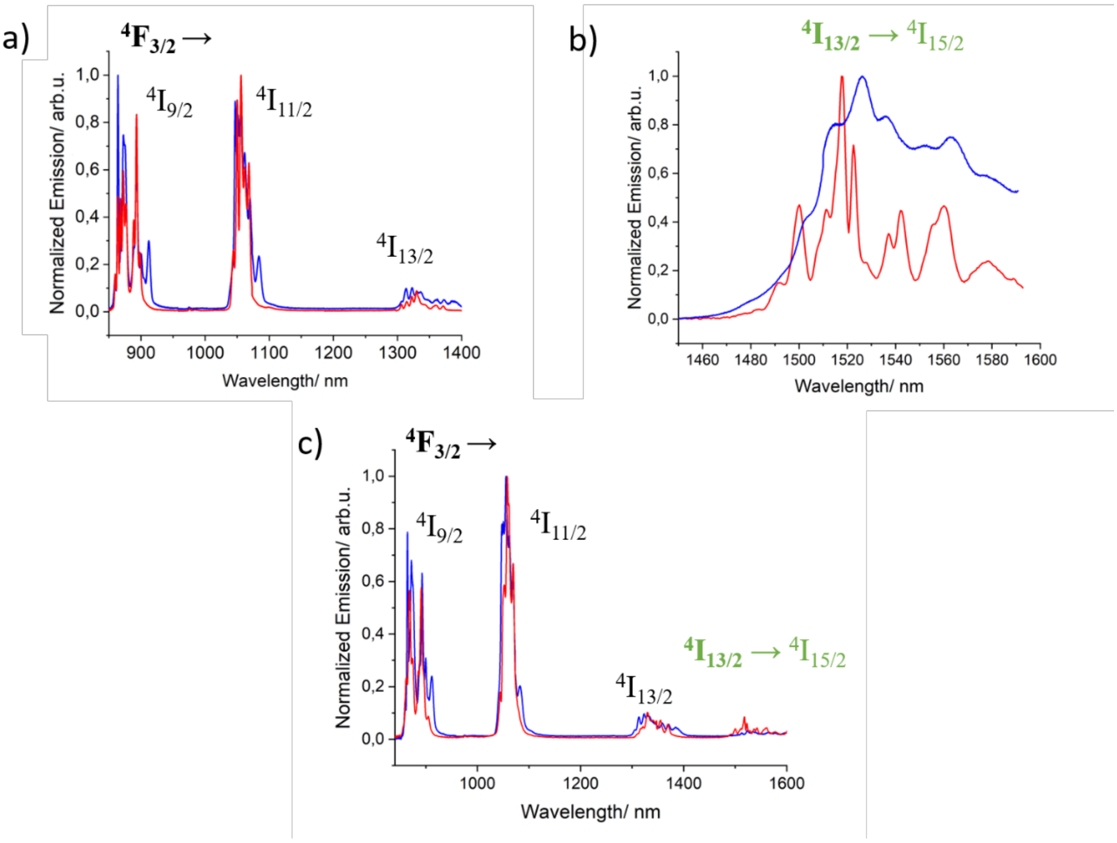

Figure S10. Normalized emission (λ_{exc}=400 nm) of [LuNdLu] (**1**) (a), [ErLaEr] (**2**) (b) and [ErNdEr] (**3**) (c) in diluted MeOD-d_4:DMSO- d_6 solution (1:1) (red trace) and the solid state (blue trace) at 77K highlighting the transitions involved for Nd^{3+} (black) and Er^{3+} (green).

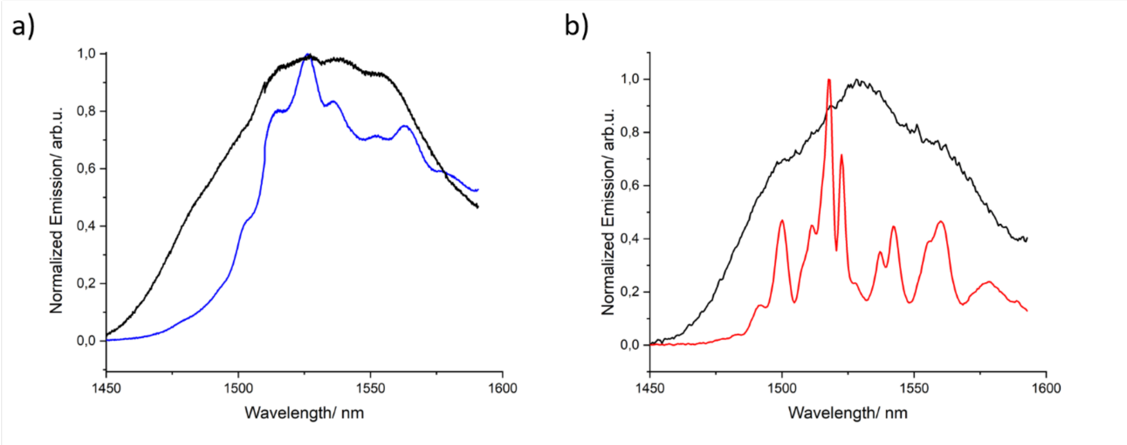

Figure S11*.* Normalized emission (λ_{exc}=400 nm) of [ErLaEr] (**2**) in the solid state (a) and in diluted MeOD-d_4: DMSO-d_6 solution (1:1) (b) at room temperature (black trace) and at 77K (blue or red trace, respectively).

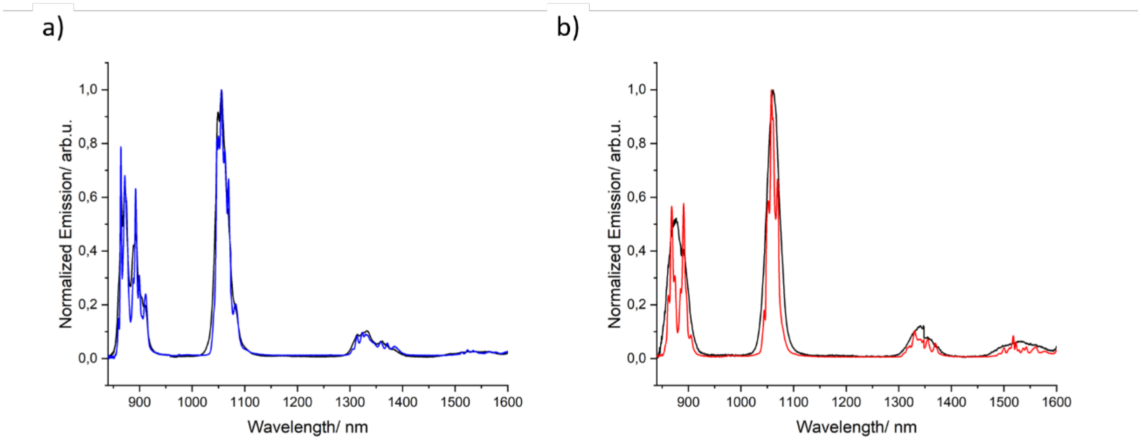

Figure S12*.* Normalized emission (λ_{exc}=400 nm) of [ErNdEr] (**3**) in the solid state (a) and in diluted MeOD-d_4:DMSO-d_6 solution (1:1) (b) at room temperature (black trace) and at 77K (blue or red trace, respectively).

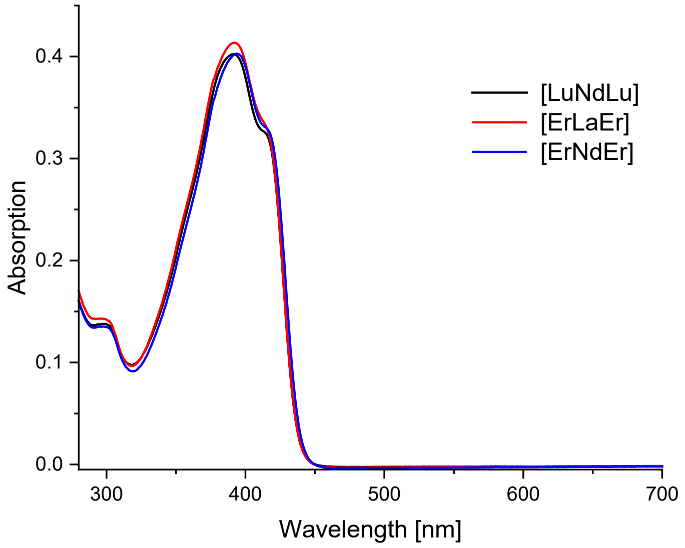

Figure S13. Absorption spectra [LuNdLu] (black trace), [ErLaEr] (red trace) and [ErNdEr] (blue trace) recorded in deuterated MeOD/DMSO solution (1/1 ratio) at room temperature.

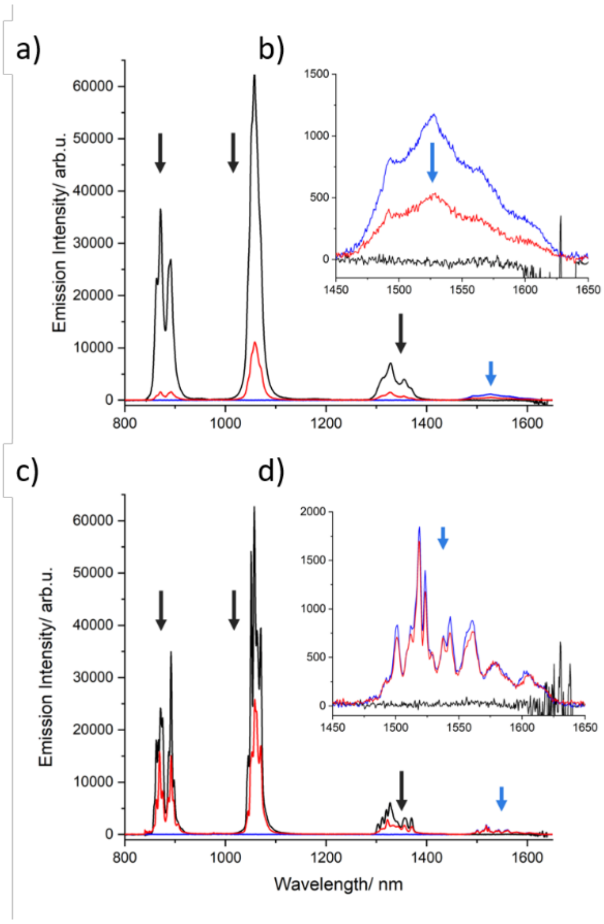

Figure S14. Emission spectra (λ_{exc} = 400 nm) of iso-absorbant solutions of complexes (**1**) (black trace), (**2**) (blue trace), and (**3**) (red trace) in MeOD-d_4: DMSO-d_6 (1:1) solution at room temperature (a) and 77K (c) with the correspondent Er^{3+} emission zoom (b and d) in both conditions.

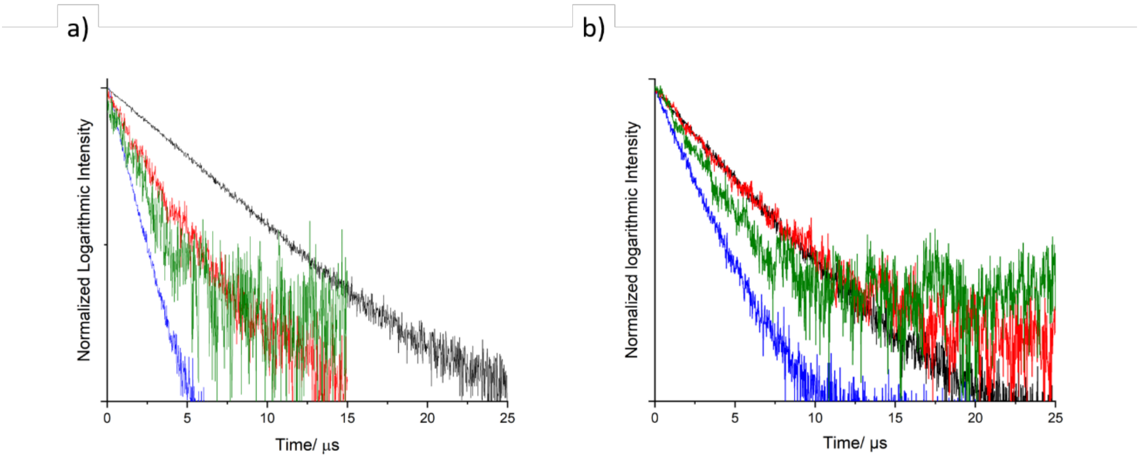

Figure S15. Excited state decay measured at 1056 nm for [LuNdLu] (**1**, black trace) and [ErNdEr] (**3**, blue trace) and at 1550 nm for [ErLaEr] (**2**, red trace) and [ErNdEr] (**3**, green trace) (λ_{exc}=400 nm) in diluted MeOD-d_4: DMSO-d_6 solution (1:1) at room temperature (a) and 77K (b).
